# Adaptive Landscape Shaped by Core Endogenous Network Coordinates Complex Early Progenitor Fate Commitments in Embryonic Pancreas

**DOI:** 10.1038/s41598-020-57903-0

**Published:** 2020-01-24

**Authors:** Junqiang Wang, Ruoshi Yuan, Xiaomei Zhu, Ping Ao

**Affiliations:** 1 Key Laboratory of Systems Biomedicine (Ministry of Education), Shanghai Center for Systems Biomedicine, Shanghai Jiao Tong University, Shanghai, China; 2 School of Biomedical Engineering, Shanghai Jiao Tong University, Shanghai, China; 3 Shanghai Center for Quantitative Life Sciences and Physics Department, Shanghai University, Shanghai, China; 4 School of Biomedical Engineering, Shanghai Jiao Tong University, Shanghai, China; 5 State Key Laboratory for Oncogenes and Related Genes, Shanghai Cancer Institute, Shanghai Jiao Tong University School of Medicine, Shanghai, China

**Keywords:** Gene regulatory networks, Dynamic networks, Dynamical systems

## Abstract

The classical development hierarchy of pancreatic cell fate commitments describes that multipotent progenitors (MPs) first bifurcate into tip cells and trunk cells, and then these cells give rise to acinar cells and endocrine/ductal cells separately. However, lineage tracings reveal that pancreatic progenitors are highly heterogeneous in tip and trunk domains in embryonic pancreas. The progenitor fate commitments from multipotency to unipotency during early pancreas development is insufficiently characterized. In pursuing a mechanistic understanding of the complexity in progenitor fate commitments, we construct a core endogenous network for pancreatic lineage decisions based on genetic regulations and quantified its intrinsic dynamic properties using dynamic modeling. The dynamics reveal a developmental landscape with high complexity that has not been clarified. Not only well-characterized pancreatic cells are reproduced, but also previously unrecognized progenitors—tip progenitor (TiP), trunk progenitor (TrP), later endocrine progenitor (LEP), and acinar progenitors (AciP/AciP2) are predicted. Further analyses show that TrP and LEP mediate endocrine lineage maturation, while TiP, AciP, AciP2 and TrP mediate acinar and ductal lineage maturation. The predicted cell fate commitments are validated by analyzing single-cell RNA sequencing (scRNA-seq) data. Significantly, this is the first time that a redefined hierarchy with detailed early pancreatic progenitor fate commitment is obtained.

## Introduction

The cell fate commitments are fundamental for understanding development and diseases, which have attracted diverse biological fields to decipher cell identities, maturation dynamics, and cell fate decision mechanisms^1–6^. Pancreas development has been extensively studied because of its close relevance to pancreatic diseases, such as diabetes, pancreatitis, and pancreatic adenocarcinoma^7–12^. Progress in pancreas development studies has made it one of the most excellent organs for understanding cell fate commitments.

There are three major mature cell types in the adult pancreas, they are exocrine acinar and ductal cells, and endocrine cells. The endocrine cells are composed of five hormone-producing cell types (α, β, δ, ε, and PP cells). Pancreas development is orchestrated by sequential cell fate commitments that finally give rise to these major mature cells^11,13^. In the mouse, pancreatic epithelial progenitor cells that have multipotency appear at around embryonic day 8.5 (E8.5)^14^, and pancreatic buds are formed by these epithelial MPs at E9.5^15^. Starting at around E12.5, the tip domain and trunk domain become visible as a result of the rapid growth of pancreatic buds. Cells in tip mature into acinar cells and cells in trunk bifurcate into ductal and endocrine cells subsequently^11^. The above lineage commitment paths are also preserved in the human pancreas^16^. Moreover, in the mouse, MPs can directly give rise to a small proportion of endocrine progenitors from E8.5 to E11.0^17^. These observations, which are mainly based on morphogenesis, are generalized as the classical hierarchy^11,13^.

Whether pancreatic MPs persist beyond E8.5 has been intensively examined. Heterogeneous progenitor cells are revealed by lineage-tracing experiments in tip domain, trunk domain and at the interface of tip and trunk in the early embryonic pancreas during E8.5-E14.5^18–20^. However, the cell potency and identity of these progenitors is insufficiently characterized^14^. Little is known about the progenitor fate commitments that how the MPs progressively give rise to acinar-committed cells or ductal- and endocrine-committed cells in the progenitor domains. One possible model is that the multipotency is determined by the concomitant expression of a set of transcription factors, such as PTF1A, SOX9, and NKX6.1, as the expression domains of transcription factors diverge, cells gradually become bi- or unipotent^14^.

With the advance of the scRNA-seq technique, adult pancreatic cells^5,21–23^, E14.5 and E17.5 pancreatic epithelial cells^24^, and E12.5 and later stage pancreatic endocrine cells^25–30^ are examined at the single-cell level. The cellular identities of progenitor cells, such as NGN3^+^ endocrine cells^25,29,30^, proliferating acinar cells^24,31^, and proliferating ductal cells^24^ are largely disclosed. And the gene expression dynamics in these cells from immature status to mature status are captured, too. However, the complete early fate commitments of pancreatic progenitors from multipotency to bi- or unipotency are not revealed yet. Moreover, though more detailed endocrine maturation paths of a hESC model are analyzed^28^, it is unknown whether these inferred multiple maturation paths represent natural maturation paths *in vivo*^28^.

Dynamic models have made progress in interpreting cell fate decision mechanisms in development from gene regulatory networks^1,2,32–38^, benefited by the merit of reflecting decision-making logic and complexities of the networks compared with statistic models^39^. The pancreatic cell fate decisions are also simulated by dynamic models^40–42^. However, modeling the pancreatic progenitor fate commitments is plagued by complex gene regulations in pancreas development. Based on the assumption that cell types are robust states evolutionarily shaped by the underlying endogenous molecular—cellular network formed by essential transcription factors, the core endogenous network hypothesis has successfully given mechanistic explanations of cell fate decisions in various cancers beyond complex regulations quantitatively^1,2,43,44^. Here we extend the hypothesis to the pancreas development process and explore the early progenitor fate commitments in embryonic pancreas. Fortunately, genetic switches and core gene regulatory circuits found in pancreas development make it possible to construct the core regulatory network. A core endogenous network for pancreatic lineage decisions was constructed based on literature references with a solid molecular basis. By quantifying the dynamic property of the network, we obtained an adaptive landscape governing the development process. Both well-characterized pancreatic cell types and previously unrecognized progenitors—TrP, LEP, TiP, AciP, and AciP2, are predicted. Moreover, complete endocrine lineage commitment path mediated by TrP and LEP, and more complex exocrine acinar and ductal lineage commitment paths mediated by TiP, AciP, TrP, and TrP2 are revealed. The predicted novel progenitors are further validated by analyzing scRNA-seq data. In conclusion, the results reveal a redefined hierarchy of early pancreatic progenitor fate commitment that has not been clarified before. Significantly, this is the first time that the detailed early pancreatic progenitor fate commitments in the embryonic pancreas are obtained.

## Results

### Construction of core endogenous network for major pancreatic lineage decisions

**Figure 1:**
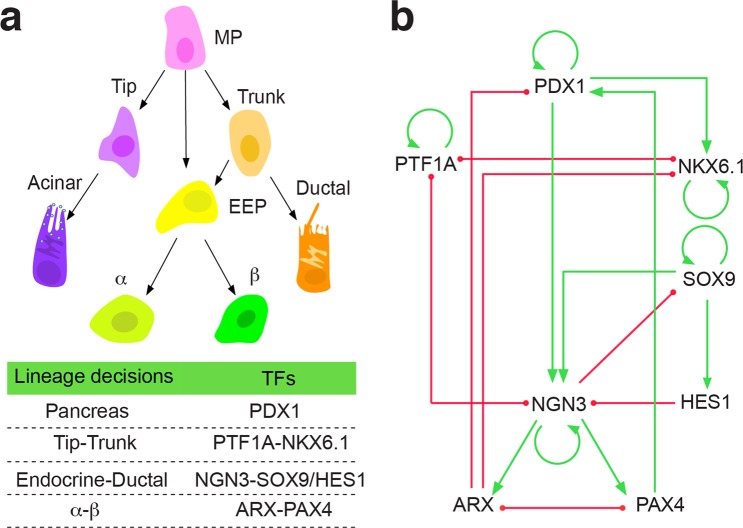
Construction of the core endogenous network for major pancreatic lineage decisions. (**a**) The classical hierarchy and master TFs determining major fate decisions. (**b**) The core endogenous network of major pancreatic lineage development. The green lines indicate up-regulations, and the red lines indicate down-regulations.

We focus on the gene regulations in embryonic pancreas from E8.5 to E14.5. Though complex gene regulations involve in pancreas development^45^, several master TFs are prominent by playing a pivotal role in determining lineage decisions (Fig. 1a). PDX1 is an essential earliest marker for pancreatic cell fate commitments, which is originally expressed in the pancreatic buds at E8.5—E9.0^46^. In PDX1^−/−^ mice, though pancreatic buds are formed, the subsequent morphogenesis is inhibited^46^. In the following lineage bifurcation process, PTF1A and NKX6.1 form a genetic switch, determining MPs adopt either ductal/endocrine-committed fate or acinar-committed fate, respectively^47^. In the trunk domain, a genetic circuit composite of NGN3-SOX9/HES1 further determines progenitors differentiating to either endocrine lineage or ductal lineage^48^. SOX9 and HES1 maintain the status of ductal cells, while NGN3 regulates endocrine cell differentiation^49,50^. SOX9 activates the expression of NGN3 and HES1. NGN3 inhibits the expression of SOX9. In the later endocrine maturation stage, an antagonistic genetic switch ARX-PAX4 operating at the downstream of NGN3 determines endocrine cell fate choices^51^. ARX specifies α cell fate, while PAX4 specifies β cell fate^51^.

These master TFs governing lineage decisions at different lineage maturation stages are not isolated, since multiple activation and inhibition regulations coordinate them. PDX1 upregulates the expression of NKX6.1^52^, as well as NGN3^53^. NGN3 further activates the expression of ARX and PAX4^51^. ARX inhibits the expression of PDX1 and NKX6.1^52^, while PAX4 promotes the program of β cell differentiation, and as a result, the expression level of PDX1 increases^54^. PTF1A and NGN3 repress the expression of each other^55^. Moreover, PDX1^56^, PTF1A^47^, NKX6.1^47^, SOX9^57^, and NGN3^58^ are self-activated, which contribute to their sustaining expressions during pancreas development. The essential interactions of these TFs identified from genetic experiments are integrated and presented in Table 1. These TFs form a closed core endogenous network (Fig. 1b) that gives a primary description of the genetic basis of pancreas lineage decisions systematically. The network structure is intrinsically different from Zhou’s network^40^. A comparison shows that Zhou’s network is constructed based on an inadequately described hierarchy of early pancreatic cell fate decisions, in which the essential tip-trunk and endocrine-ductal lineage bifurcations are not present. Consequently, master TFs together with the accompanying regulations dominating the above bifurcations are missing in Zhou’s network. Since cell phenotypes emerge from the dynamics of regulatory networks, the constructed core endogenous network enables us to further decipher cell fate commitments in early pancreatic development using a coarse-grained model.

**Table 1:** Activation and inhibition relationships of the essential TFs in the core endogenous network for major pancreatic lineage decisions.

TFs	Activators	Inhibitors
PDX1	PAX4^81^; PDX1^56^	ARX^52^
PTF1A	PTF1A^47^	NKX6.1^47^; NGN3^55^
NKX6.1	NKX6.1^47^; PDX1^52^	PTF1A^47^; ARX^52^
SOX9	SOX9^57^	NGN3^48^
HES1	SOX9^48^	
NGN3	PDX1^53^; SOX9^48^; NGN3^48,58^	HES1^48^; PTF1A^55^
ARX	NGN3^51^	PAX4^51^
PAX4	NGN3^51^	ARX^51^

Eight master transcription factors determining pancreas cell fates during pancreatic development are included. There are 12 activation and 10 inhibition relationships. SOX9-FGF positive feedback loop contributes to the self-activation of SOX9 expression^57^, and NGN3-MYT1 positive-feedback loop^58^ contributes to the self-activation of NGN3 expression. These loops are represented by the self-activation loops.

In the network, the regulatory relationships are robustly preserved, which means the expression status of each gene uniformly and significantly affects the expression of its target genes. The model does not include the activation of PDX1 by PTF1A and the inhibition of ARX by NKX6.1, considering these two interactions do not show uniformity and significance in regulation. PTF1A is reported to active the PDX1 promoter at relatively early stage^59^, but the activation does not work later. Similarly, the proposed inhibition of ARX by NKX6.1 during endocrine development^52^ becomes ineffective in β cells^52^. Moreover, PTF1A is not necessarily required for PDX1 expression^60^, which weakens the significance of the corresponding regulatory relationship. We also simulated the networks including these two regulations, results from which imply that they are not significant in cell fate commitments, which is discussed later.

### Quantification of the intrinsic equilibrium states and the topological adaptive landscape emerging from the core network dynamics

In the development processes, the expression statuses of these TFs in the core network are dynamic. However, since feedbacks coordinate their expression, only limited states of the network can reach equilibrium, that is, the expression statuses of these TFs are balanced, which do not change without external force. Mathematically, these states are called equilibrium points in dynamical systems. The equilibrium states can be further classified into stable states, transition states, or hyper-transition states based on the eigenvalue characteristics of their Jacobian matrices. Stable states usually represent stable cell types, and small perturbations can not cause cells escaping from these states. While transition and hyper-transition states represent intermediate cellular states that mediate spontaneous transitions, where small perturbations can trigger cells escaping to their connecting stable states.

**Figure 2:**
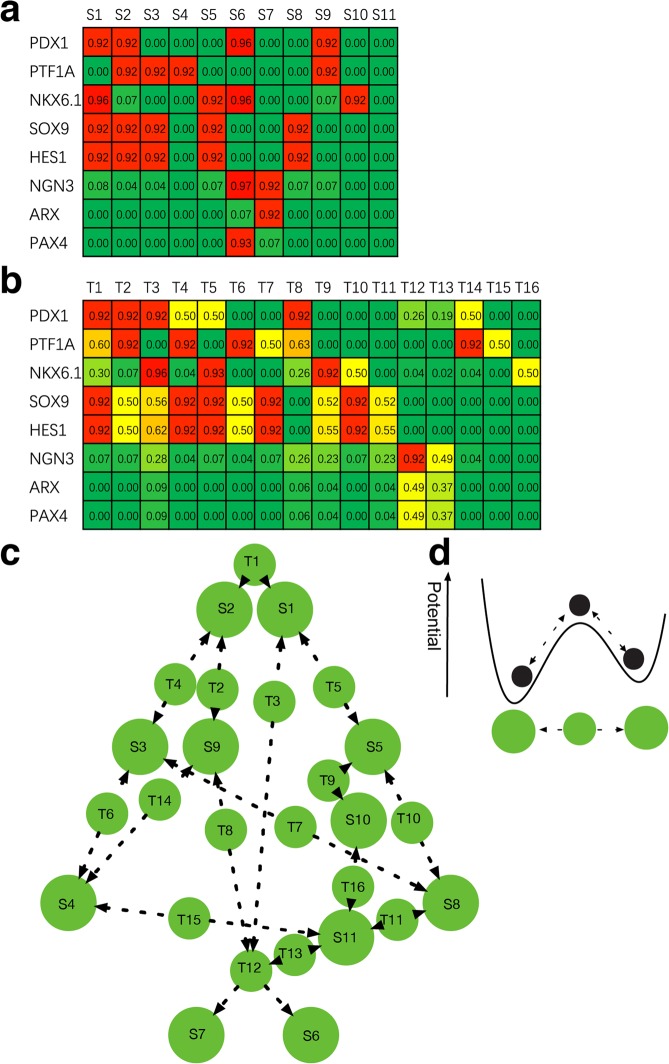
Stable states, transition states, and their transition relationships on the adaptive landscape. (**a**) Stable states in the core network. Each column represents one stable state. Each row represents the expression statuses of one TF. “1” represents the maximal expression, and “0” represents no expression. (**b**) Transition states in the core network. (**c**) Transition relationships of stable states and transition states on the adaptive landscape. The large circles represent stable states, while the small circles represent transition states. The arrows indicate transition directions induced by small perturbations. (**d**) Potential illustration of the landscape. Stable states are envisaged as valleys with low potential energy, which are insensitive to small perturbations. Transition states are envisaged as saddles with higher potentials connecting the neighboring wells. The arrows indicate the transitions are reversible under stochastic noise.

To quantify the equilibrium states of the network, we transfer the regulatory network into a set of ordinary differential equations (ODEs) using a coarse-grained method as described in the method section. This method has been examined in a variety of studies^38,43,44,61,62^. We obtained 11 stable states (Fig. 2a), 16 transition states (Fig. 2b) and 8 hyper-transition states (Supplementary Fig. S1) in the network under the parameter *n* = 4. We also obtained their transition relationships (Supplementary Fig. S2**)** by performing the perturbation analysis (see methods). The adaptive landscape can give a vivid representation of the multistability property of complex biological systems^63–65^. To briefly visualize the multistability property, we represented the transition relationships mediated by transition states on a topological adaptive landscape (Fig. 2c). Because hyper-transition states connect multiple stable states, either lineage conversions or direct differentiations are mediated by these states. We don’t consider the above situations. On the landscape, the stable states are viewed as valleys with locally lowest potentials, and the transition states are viewed as saddles connecting these valleys (Fig. 2d). The development processes are viewed as cell jumps among these valleys separated by saddles, with valleys representing stable cell phenotypes and saddles representing intermediate cell phenotypes.

Further, we tested the robustness of the multi-stability feature of the network against different parameters and models. The activity of the core TFs may vary during development, considering there are fluctuations in the concentrations of co-regulators of these TFs. The parameter *n* in the ODE model, determining the steepness of the Hill-equation, can reflect the catalyzing kinetics of the biochemical reactions. Thus, we obtained the equilibrium states under different parameters (*n* = 5–7) (Supplementary Figs. S3–S5). All the stable states and most transition/hyper-transition states found under *n* = 4 are preserved, with only slight alterations in values. This reveals that these intrinsic states robustly exist for a wide range of parameters. Moreover, we used an alternative model, the Boolean network model, which is less dependent on parameters, to obtain stable states by enumerating all possible initial states (Supplementary Fig. S6). All the stable states found in the ODE model are reproduced in the Boolean network model since at each stable state they show the same expression patterns. The consistency of the results obtained from these two different models further verifies that these stable states are an intrinsic robust dynamic property.

### Both well-characterized pancreatic cells and previously unrecognized progenitors are predicted by the intrinsic equilibrium states

**Figure 3:**
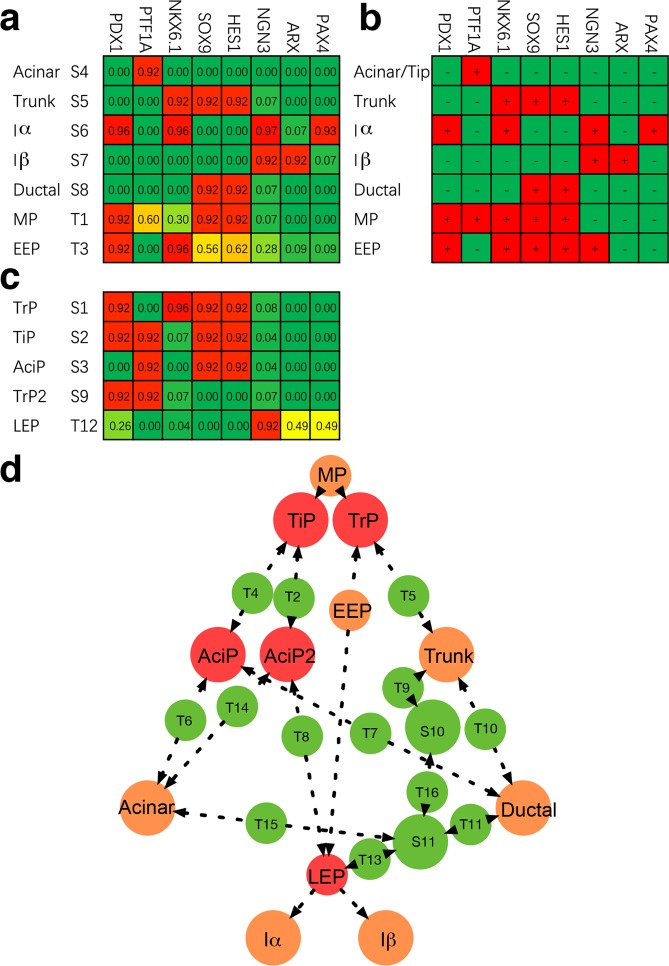
Pancreatic cell types predicted by the core endogenous network and their positions on the adaptive landscape. (**a**) The well-characterized cell types reproduced from the core network. Each row represents one cell type. Abbreviations: Iβ, immature β; Iα, immature α; MP, multipotent progenitor; EEP, early endocrine progenitor. (**b**) Experimental observations of TF expression statuses in the well-characterized cell types. “ + ” represents gene expression “on”, and “−” represents gene expression “off”. Considering E12.5—E14.5 cells in tip and trunk are heterogeneous and progenitor cell types in these domains are not well characterized, the tip cell and trunk cell here represent the well-characterized cell types in tip and trunk domain. Acinar and tip cells have the same core TF expression statuses. References are shown in Supplementary Table 1. (**c**) Previously unrecognized progenitor cell types predicted from the core endogenous network. Abbreviations: TrP, trunk progenitor; TiP, tip progenitor; AciP, acinar progenitor; AciP2, acinar progenitor 2; LEP, later endocrine progenitor. (**d**) The predicted cell types on the adaptive landscape. Previously unrecognized cell types our model predicted are colored by red, well-characterized cell types are colored by orange, and unclassified states are colored by green.

Since equilibrium states usually represent stable or intermediate cell types, we further examined whether these quantified states represent pancreatic cell types. The well-characterized acinar/tip, trunk, immature α (Iα), immature β (Iβ), and ductal cell types are captured by stable states S4–S8 separately (Fig. 3a,b). These cell states are exactly consistent with the corresponding cell types in TF expression statuses. Moreover, two transitory cell types are captured by transition states (Fig. 3a,b). State T1, which shows an expression pattern the same as MP cells, is identified as a MP state. State T3, which shows an expression pattern the same as the early endocrine progenitor, is identified as an early endocrine progenitor (EEP) state. These well-characterized pancreatic cell types are exactly reproduced from the core endogenous network.

More interestingly, some progenitor cell types that previously unrecognized are predicted by other states (Fig. 3c). Two stable states S1 and S2 express early pancreatic progenitor differentiation markers^28^ SOX9, HES1, and PDX1, but with PTF1A and NKX6.1 exclusively expressed. These two states have expression patterns similar to MP, indicating their differentiation statuses are less mature than the tip or trunk cells. They are predicted as tip progenitor (TiP) state and trunk progenitor (TrP) state, separately. State S3, which not only expresses PTF1A but also expresses progenitor markers SOX9 and HES1, is predicted as an acinar progenitor (AciP) state. The AciP state also resembles the centroacinar cell, a rare cell type that is marked by SOX9 expression^66^. State S9 expresses PDX1 and PTF1A. Noting that PTF1A^+^PDX1^+^CpaI^+^cMyc^HI^ cells are retained in the differentiated acini^20^, S9 may represent acinar progenitor cells as well and is marked by AiP2. Sate T12, which connects EEP, Iα, and Iβ, is predicted as a later endocrine progenitor (LEP) state. Two states S10 and S11 are not classified. S10 only expresses NKX6.1 and S11 expresses none of the major cell markers, which lack the marker characters of progenitor cells of all the three cell lineages, indicating they are not progenitor cell states belonging to these three cell lineages. Moreover, different from other predicted cell states, these two states have no interconnections within none of the three cell lineages (Fig. 2c). The topological connection also reveals that they do not contribute to the progenitor cell fate commitments of the three major cell lineages. In all, besides reproducing the well-characterized cell types, the core endogenous network also predicts a set of pancreatic progenitors that are not recognized previously.

To check whether the activation of PDX1 by PTF1A and the inhibition of ARX by NKX6.1 affect the above cell states, we simulated the network including these two regulations. Except for the AciP state and the acinar state, all the other predicted cell types are reproduced from the network at the existence of the activation of PDX1 by PTF1A (Supplementary Fig. S7). The altered network can not give a very correct prediction since the known acinar state can not be produced, supporting the activation does not work at the later development stages. All the predicted cell states are preserved at the existence of the inhibition of ARX by NKX6.1 (Supplementary Fig. S8), indicating the inhibition does not affect these cell states. Though we can not deny the existence of these two interactions, the results from model simulation imply these two interactions do not play significant roles in cell fate commitments.

Finally, we mapped the inferred cell types to the topological adaptive landscape (Fig. 3d). On the landscape, the topological connections of the already known cell types reveal the same fate commitment order as that in the classical hierarchy. However, previously unrecognized progenitors the model predicted also lie on the lineage maturation paths, indicating more complex early cell fate commitments.

### The previously unrecognized TrP and LEP cells mediate the early endocrine lineage commitment path

**Figure 4:**
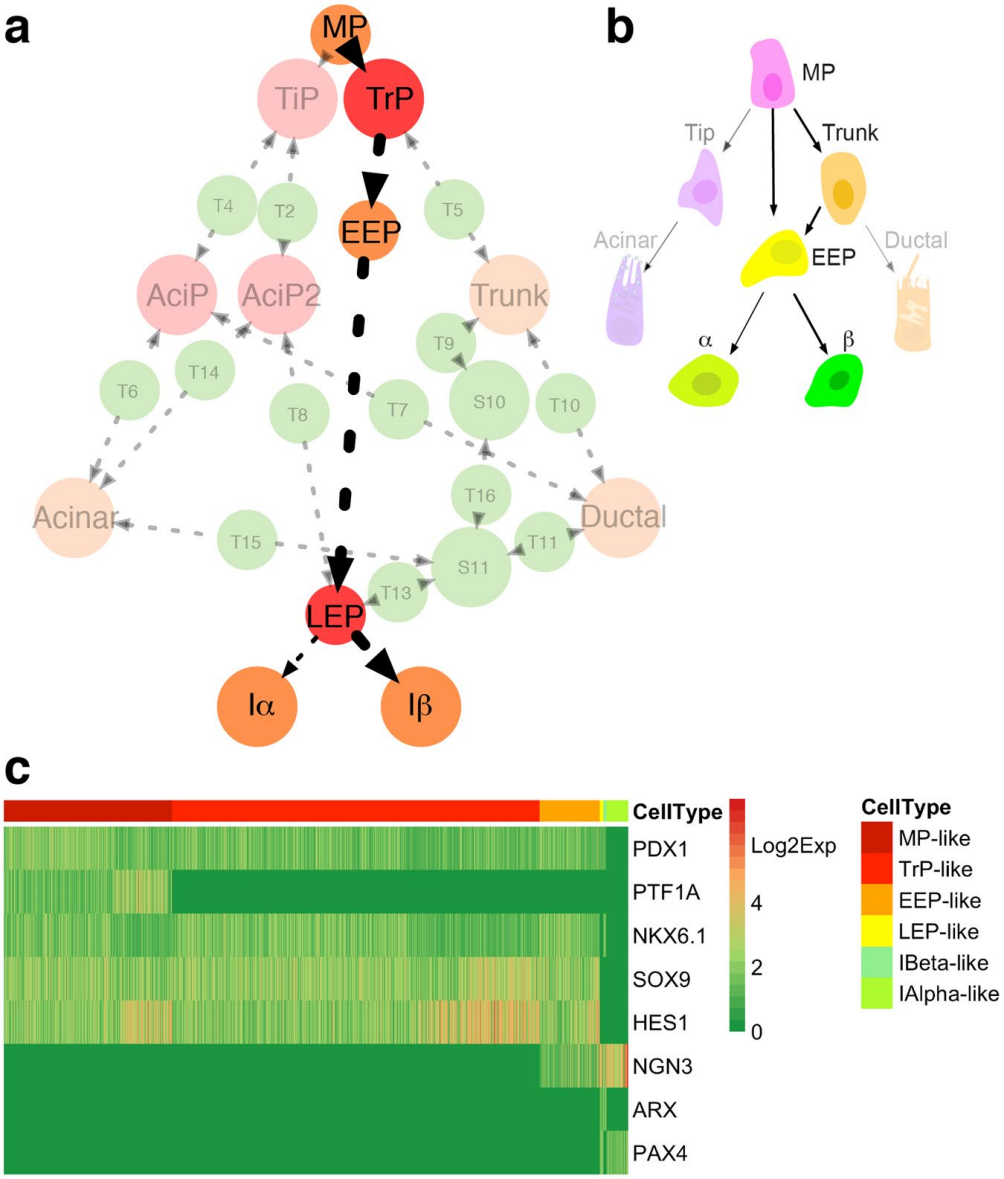
The predicted endocrine lineage commitment path mediated by TrP and LEP, and validation of the predicted endocrine lineage progenitors. (**a**) The progenitor fate commitment path of endocrine lineage is revealed from the topological adaptive landscape. The previously uncharacterized progenitors TiP and LEP are colored by red. The known cells are colored by orange. The path is highlighted by thick lines, with the arrows indicate maturation direction. Cells at Iα and Iβ sates will turn into mature α and mature β cells subsequently. (**b**) Graphical depiction of the endocrine maturation paths in the classical hierarchy. The paths in (**a**) and (**b**) are highlighted by thick lines, with different bold types indicating different fates either to Iα state or to Iβ state. The other irrelevant cell types are made transparent. (**c**) Validation of the predicted endocrine lineage progenitor cell types. All the predicted endocrine progenitors are reproduced from the datasets. Cells show exact expression patterns the same as the predicted progenitor cells are shown.

We then examined the endocrine lineage commitments on the adaptive landscape. A path with multiple differentiation stages mediated by the previously unrecognized TrP and LEP cells is predicted (Fig. 4a). Along the path, MPs first differentiate into TrP state, then go across EEP state and LEP state, and finally bifurcate into Iα state and Iβ state. Compared to the classical model (Fig. 4b), The predicted path reveals complete early endocrine fate commitments that have not been clarified. Firstly, the EEP and the trunk cells do not mature directly from the MP cells but the previously unrecognized TrP cells. Secondly, the LEP cells, not the EEP cells, are the direct progenitors of Iα and Iβ cells.

To validate the predicted endocrine fate commitments, we analyzed the scRNA-seq data of murine embryonic pancreas^24^. We merged all the E12.5, E14.5 and E17.5 cells and then examined the expression statuses of the core TFs from the whole dataset. Very interesting, not only the previously well-characterized MP, EEP, Iα, and Iβ cells are reproduced, but also the TrP and the LEP cells are revealed (Fig. 4c). Other major cell markers that are reported indicating the cellular identities of pancreatic epithelial cells^24^ are shown as well (Supplementary Fig. S9). BTBD17 is highly expressed in NGN3^high^ LEP-like, Iα-like, and Iβ-like cells. The result is consistent with the previous report that BTBD17 is a NGN3^+^ cell marker^24^. Moreover, we find that BTBT17 has a relatively low expression level in NGN3^low^ EEP-like cells.

To check whether these progenitor cells are dominant in the endocrine and ductal progenitor population, we further analyzed the NKX6.1^+^ cell population since endocrine and ductal cells are matured from NKX6.1^+^ progenitors^47^. Excluding a considerable proportion of PDX1 and NKX6.1 expressing mature β cells, cells harboring the expression pattern similar to the progenitor cells our model predicted occupy the majority in the NKX6.1^+^ cell population (Supplementary Fig. S10).

We also analyzed the E17.5—P60 (postnatal day 60) mouse islet α/β scRNA-Seq data^29^. Their expression patterns at the core network level are given (Supplementary Fig. S11a). Except that a small proportion of NGN3^+^ cells display expression patterns similar to Iα-like or EEP-like cells (Supplementary Fig. S11b), most of the cells display expression patterns similar to mature endocrine α or β cells. The data give rare information on early endocrine cell fate commitments. We speculate the main reason is that most of the early pancreatic fate commitments occur during E9.5—E14.5, which are earlier than the time points that the cells are sequenced.

### Deciphering endocrine lineage commitments in a hESC model

**Figure 5:**
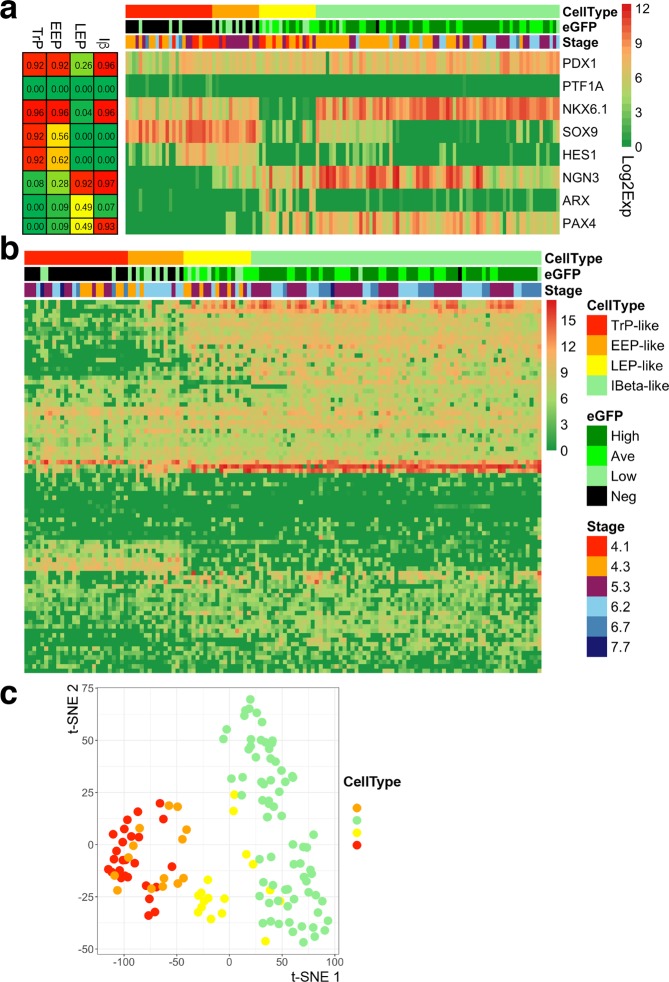
Validation of the predicted TrP and EEP cells and endocrine β lineage commitments in the hESC model. (**a**) Validation of the predicted TrP and EEP states in the hESC model. In the hESC model, a 7-stage differentiation protocol and a NEUROG3-EGFP hESC line were used. The EGFP was expressed under the control of endogenous NEUROG3 locus. TrP and LEP states are found from the heterogeneous endocrine cells. EEP and Iβ states are reproduced, as well. TrP and EEP cells express no or few EGFP, indicating the immature statuses of these progenitors. The differentiation stages from stage 4.3 to stage 7.7 they have indicate that they do not mature drastically. (**b**) Broad gene expression profiles of these inferred cell types. (**c**) The plot of the first two t-SNE components of the gene expression.

Endocrine β cell maturation paths at the single-cell level are examined in a hESC model, and multiple paths are proposed to explain β cell maturation^28^. However, the relationships of these paths to the nature maturation path *in vivo* are unknown. Here we re-analyzed the endocrine single-cell gene expression data of the hESC model. Very interestingly, the predicted progenitors TrP, EEP, LEP and Iβ are detected (Fig. 5a). These cell types reveal distinct expression profiles at a broad level (Fig. 5b). This indicates that the expression patterns at the core network level are reliable indicators of the cellular maturation status. Further, we use the dimensionality reduction method t-distributed stochastic neighbor embedding^67^ (t-SNE) to visualize the data. The first two t-SNE components of these cell types display gradual change along the maturation path (Fig. 5c). The result shows the natural mature path our model predicted, which has not been completely revealed by any of the proposed paths^28^, exists in the hESC model.

**Figure 6:**
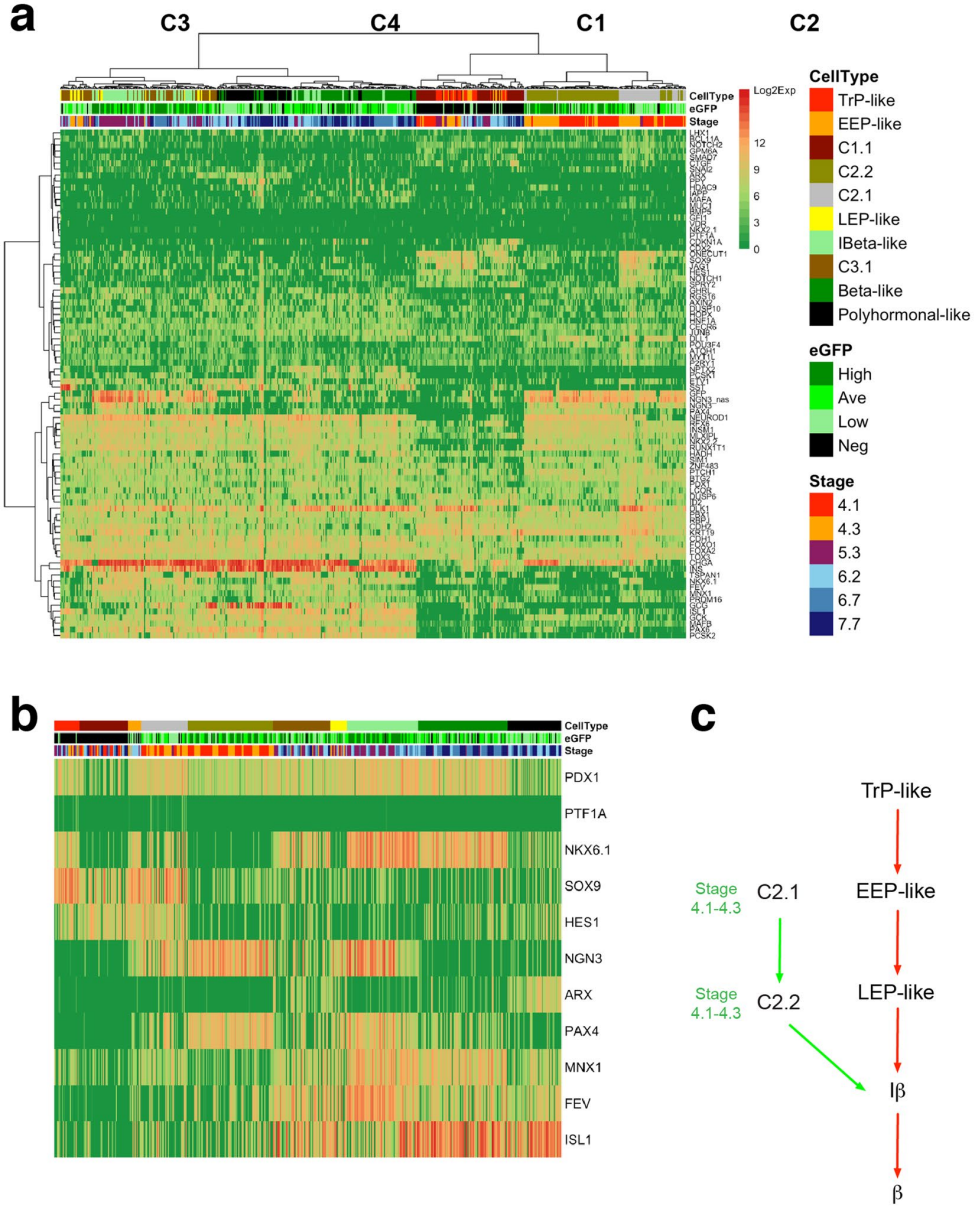
Deciphering the complex β cell fate commitment paths in the hESC model. (**a**) Heatmap indicating the transcription similarity of the cells. The correlation distance was used here, and four major groups (C1–C4) were clustered. Cells in each group are further divided into different subgroups based on the expression statuses of TFs in the core network, as are shown in (**b**). (**b**) Expression statuses of TFs in the core network and other cell markers. Cells in the C1.1 group sporadically express PDX1 in low level and do not express NKX6.1, which is different from TrP-like and EEP-like cells. Cells in the C3.1 group are different from LEP-like cells, which express high NKX6.1. Cells in C2.1 show less maturation status than C2.2, since they express progenitor cell markers SOX9 and HES1. (**c**) Two main independent endocrine lineage maturation paths are inferred. The red arrows indicate the natural maturation path predicted by our model, while the green arrows indicate the atypical maturation path.

Further, we reconstructed the additional maturation paths in the hESC model under the guide of our model prediction. To measure the expression similarities of different cells in the dataset, the heatmap was generated (Fig. 6a). Four major groups (C1–C4) were clustered, and cells in each group were further divided into subgroups based on the expression statuses of TFs in the core network (Fig. 6b). Since makers MNX1, FEV, and ISL1 also indicate cellular maturation statuses^24,28^, they are presented here as well (Fig. 6b). Cells in C2.1 and C2.2 group have a very close distance to TrP-like and EEP-like cells, and exist at very early stages (stage 4.1–4.3), indicating they are early progenitor cells. A considerable proportion of eGFP^-/low^ cells in C3.1 express polyhormonal marker ARX, indicating they have adopted to polyhormonal cell fate. In addition to the path predicted by our model, an independent maturation path comprised of C2.1 and C2.2 cells is naturally revealed (Fig. 6c). This path overlaps with the previously predicted path marked by the dynamic change of NKX6.1and MNX1^28^. Because this path has no counterpart on the adaptive landscape, which should be an abnormal path that does not exist in the natural pancreas embryonic developmental processes *in vivo*. The previous result^24^ from pseudotime ordering of the murine pancreas single cells also shows that there is only one maturation path in the embryonic pancreas, in which PAX4^+^ cells arise later than NGN3^+^ cells, which matches the natural maturation path our model predicted. However, in the abnormal path compose of C2.1 and C2.2 group cells, NGN3 and PAX are expressed almost at the same time. The result also supports that the abnormal path is absent in the normal development.

### Complete early fate commitment paths of exocrine acinar and ductal lineage are predicted from the adaptive landscape

**Figure 7:**
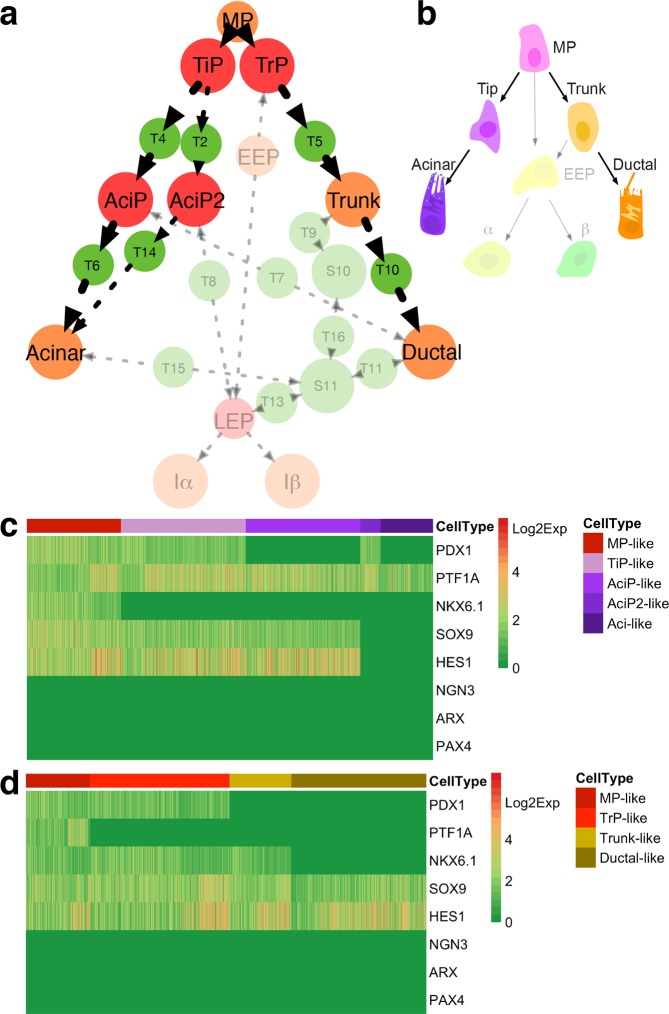
The inferred fate commitment paths of exocrine acinar/ductal lineages, and validation of the predicted cell types. (**a**) Complete exocrine acinar and ductal lineage cell fate commitment paths are revealed from the topological adaptive landscape. The previously unrecognized progenitors our model predicted are colored by red. Already known cell types are colored by orange. Two acinar lineage differentiation paths and one ductal lineage differentiation path are predicted, with arrows indicating maturation directions. (**b**) The classical fate commitment paths of exocrine acinar and ductal lineages. The irrelevant cell types are made transparent. (**c**) Validation of the predicted acinar lineage cell types. All of the predicted acinar lineage cells are reproduced from the dataset. (**d**) Validation of the predicted ductal lineage cell types. All of the predicted ductal lineage cells are reproduced from the dataset. In the above heatmaps, cells show exact expression patterns the same as the predicted progenitor cells are shown.

We then deciphered the cell fate commitments of exocrine acinar and ductal lineages. On the adaptive landscape, the progenitors TiP, AciP, and AciP2 our model predicted bridge the MP state and the acinar state (Fig. 7a). Accordingly, two independent acinar differentiation paths are revealed. One path is mediated by TiP and AciP states, while the other path is mediated by TiP, and AciP2 states. Interestingly, the previous study shows that the centroacinar cells can give rise to acinar cells^68^. The first path possibly explains the above lineage relationship as well, considering the AciP state resembles the centroacinar cells. In the ductal lineage, the MPs firstly differentiate into TrP cells, then differentiate into trunk cells, and finally mature into ductal cells. Different from the classical maturation path (Fig. 7b), the predicted paths show additional layers caused by these previously unrecognized progenitors.

We validated our model predictions using the same murine pancreatic dataset^24^. All the acinar lineage cell states our model predicted are exactly reproduced from the dataset at the core network level (Fig. 7c). A large proportion of cells show AciP-like pattern, not AciP2-like pattern, indicating the path mediated by AciP state are more frequently to be adopted than the path mediated by AciP2 state. The whole ductal lineage cell states are reproduced as well (Fig. 7d). Moreover, we checked the expression statuses of other exocrine cell makers^24^. REEP5 and TMEM97 are highly expressed in MP-like, TiP-like, AciP-like, AciP2-like, and acinar-like cells, and SPP1 is highly expressed in TrP-like, trunk-like, ductal-like cells, and a very small proportion of MP-like cells (Supplementary Fig. S9). The result is consistent with the previous report that REEP5 and TMEM97 are highly expressed in proliferating acinar cells and mature acinar cells, and SPP1 is highly expressed proliferating ductal cells and mature ductal cells^24^. Besides, our results showed that these cell markers are also expressed in progenitors that have not loss multipotency or bipotency. Previously, we verified NKX6.1^+^ progenitor cells our model predicted are dominate in the NKX6.1^+^ cell populations. To validate whether the predicted acinar lineage cells are also dominant in the cell population, we further analyzed the PTF1A^+^ cell population since acinar cells are matured from PTF1A^+^ progenitors^47^. Cells harboring the expression pattern similar to these progenitor cells our model predicted also occupy the majority in the PTF1A^+^ cell population (Supplementary Fig. S12).

The predicted acinar lineage cell states are further compared with the adult acinar scRNA-seq data^31^ (Supplementary Fig. S13). The reported proliferating SOX9^+^STMN1^+^ acinar progenitor-like cell has the same expression pattern as AciP cell, indicating they are probably the same cell type. The acinar state is reproduced as well. Moreover, the acinar cells and the acinar progenitor-like cell differ at the genomic expression level^31^ (Supplementary Fig. S14), indicating the TFs in the core network are reliable cell markers. The MP, AciP2, and TiP states are not found. Since pancreatic progenitors disappear in later development stages^20^, it is not surprising that these progenitors are not found in the adult acinar dataset.

### Simulation of the stochastic maturation dynamics by most probable paths

**Figure 8:**
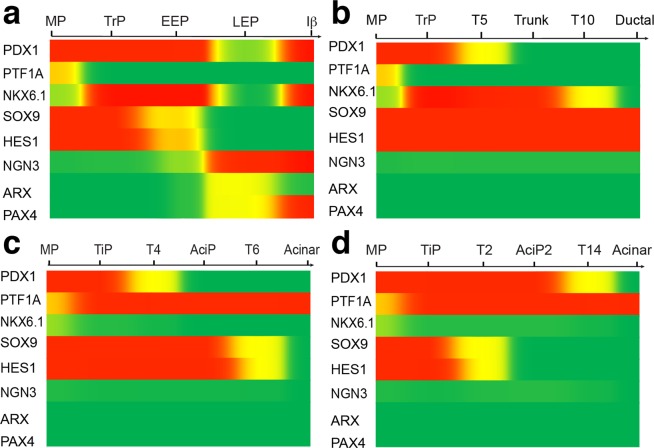
The MPPs of the major lineage maturation. (**a**) The MPP of endocrine β lineage maturation. (**b**) The MPP of ductal lineage maturation. (**c** and **d**) The MPPs of the acinar lineage maturation.

Transitions among cell states can be driven by noises (an illustration of the transition is shown in Fig. 2d), such as transcriptional noises and fluctuating signals^69,70^. Moreover, transitions are not arbitrary in the stochastically perturbed dynamical systems. The most probable path (MPP), or termed least action path, facilitates the transition by minimizing the energy cost along the transition path^71,72^. We predicted the dominant TF dynamics of the lineage maturations by the MPPs. Here the MPPs were obtained under A-type integration^72^ (see Methods). Since the real biochemical parameters are unknown, the MPPs just give raw estimations of the developmental dynamics. However, the MPPs give continuous dynamic regulatory patterns. Compared to the static expression patterns in endocrine lineage progenitors (Fig. 4c), more detailed non-monotonous TF expression dynamics in β cell lineage maturation are revealed (Fig. 8a). PDX1 and NKX6.1 drastically decrease during the transition from EEP to LEP, and further increase during the transition from LEP to Iβ. The MPPs of the acinar and ductal lineages are given, too (Fig. 8b–d). In the acinar lineage maturation, distinct dynamics along two different paths are revealed (Fig. 8c,d).

Probabilistically, other transition paths that are not mediated by the progenitor states our model predicted should occur at rare frequency, which is consistent with the fact that in the scRNA-seq data of murine pancreas development, cells harboring features do not resemble the progenitor states our model predicted are rare in the progenitor cell population. Besides the MMPs, transition paths mediated by the hyper-transition states may also have significant biological meanings. At the existence of large stochastic noise, these transition paths, passing through which higher energies are required, can be activated^73,74^, which may explain rare lineage conversions or direct maturations, as implied by their topological connections to the stable states. However, intense deterministic stimulations of specific TFs in the core network by exogenous agents can also trigger the cells deviating from the MPPs. Considering the maturation of the pancreatic cells induced by culture in the hESC model is exactly in the same situation, the abnormal maturation path composed of C2.1 and C2.2 possibly arise in this way. Though both extra cell types arise in the above situations, the extra cells generated by the former are comparatively rare in the whole population, which gives a criterion to identify whether they arise from noise or hidden regulations outside the core network.

## Discussion

We addressed the fundamental issue in pancreas development—the early progenitor fate commitments that ensure pancreatic lineage maturation in sequential order to distinct functional cell types. Complex early pancreatic progenitor fate commitments are predicted from the dynamics of a core endogenous network, which not only elucidate the identities of previously unrecognized progenitor but also reveals their lineage relationships. These predictions are further validated by scRNA-seq data. Our results reveal that the expression patterns of the TFs in the core network well characterize the progenitor identities in early pancreas development. This supports the previously proposed hypothesis that the gradual loss of pancreatic progenitor multipotency is associated with divergent concomitant expression of a set of transcription factors^14^. A clear cell fate commitment hierarchy is further revealed by our model.

**Figure 9:**
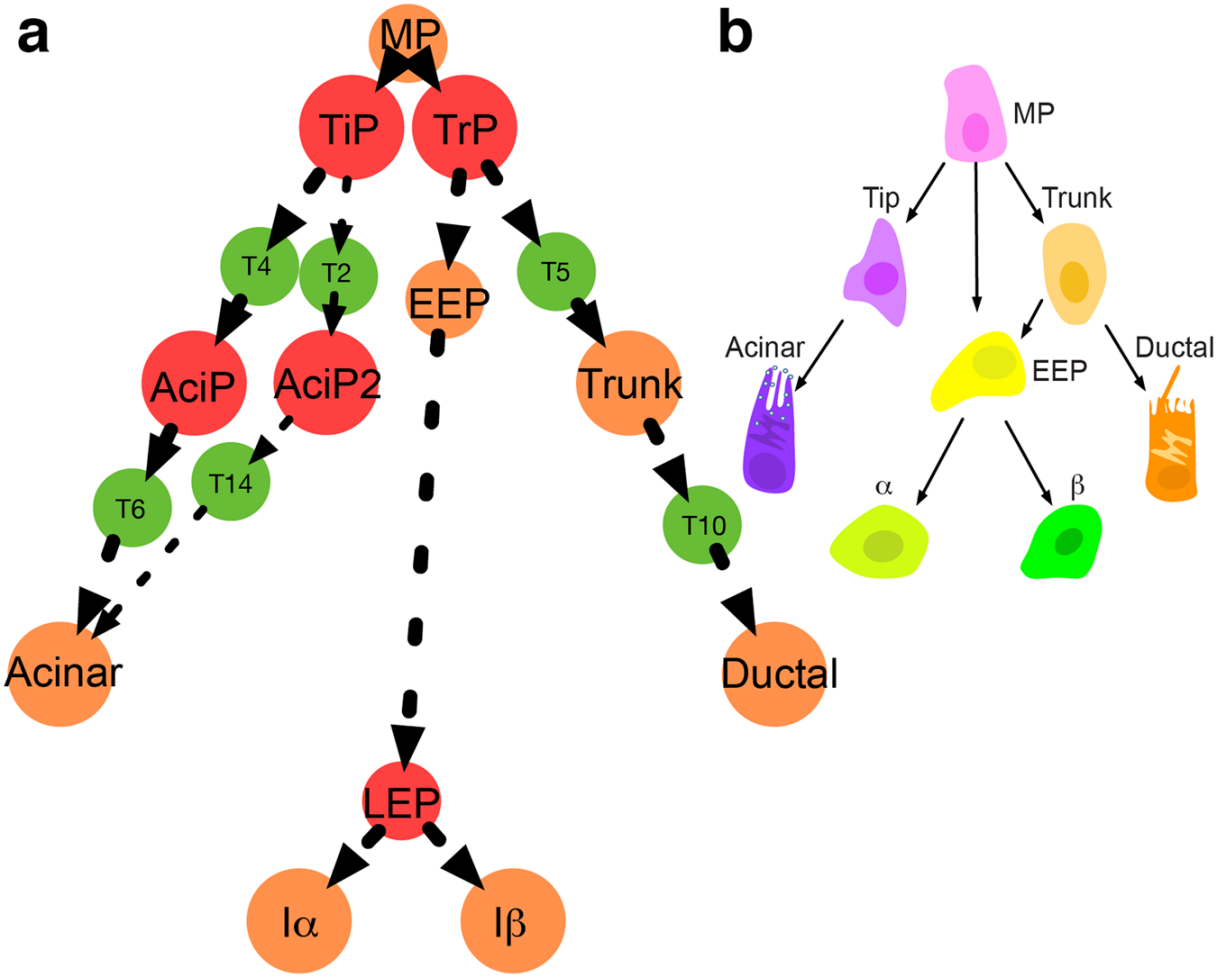
A comparison of fate commitment models in major pancreatic lineages. (**a**) Redefined cell fate commitment model. The previously uncharacterized progenitors TiP, AciP, AciP2, TrP, and LEP are colored by red. The known cells are colored by orange. The redefined model shows that these previously uncharacterized progenitors mediate the lineage maturation in the early embryonic pancreas. (**b**) The classical hierarchy.

A redefined pancreatic fate commitment model is given (Fig. 9a). The redefined model challenges the classical hierarchy (Fig. 9b). Firstly, diverse previously unrecognized progenitors including TiP, AciP, AciP2, TrP, and LEP are predicted in the redefined model. Since lineage tracing experiments show that progenitors at tip and trunk domains are heterogeneous and are not well characterized^19,20^, it is likely that the progenitors TiP, AciP together with MP and acinar cells reside in the tip domain, while the progenitors TrP, EEP together with MP and trunk cells reside in the trunk domain. Secondly, these predicted progenitors involve in the early fate commitments of pancreatic lineages. The TrP and LEP mediate endocrine lineage maturation, the TiP, AciP, and AciP2 mediate acinar lineage maturation, and the TrP mediates ductal lineage maturation. In all, the redefined model unravels a more detailed early progenitor fate commitment hierarchy of three major pancreatic lineages.

Though pancreatic single-cell data have been extensively analyzed, the whole progenitor fate commitments from multipotency to bi- or uni-potency in the embryonic pancreas are not disclosed before. We validated our model predictions by analyzing previously published datasets. Early progenitor fate commitments in endocrine, acinar, and ductal lineages our model predicted are completely verified from the murine pancreatic cell dataset. Moreover, we decipher the endocrine lineage commitments in a hESC model. Besides the natural maturation path, an atypical maturation path in the hESC model which does not naturally exist in the embryonic pancreas is also revealed. We also analyzed an adult acinar cell dataset and an E17.5—P60 stage α/β cell dataset. Only a small set of unipotent progenitor-like cells, such as AciP-like, Iα-like, and EEP-like cells are found. No MPs or bipotent TiP or TrP cells are found in these two datasets. The result is consistent with the fact that multipotency or bipotency loses at later developmental stages^14^.

The core endogenous network approach shows its powerful predictive capacity in deciphering cell fate commitments emerging from the dynamics of core regulatory networks. Not only well-characterized pancreatic cells but also previously unrecognized progenitors are accurately predicted by our model. The predicted cell states are repeatedly found in different single-cell data and reliably characterize the maturation statuses of pancreas cells at the core network level. Notably, the prediction is independent of the scRNA-seq analysis since no information from the single-cell data is used. The core endogenous network approach for development has practical significance, which is applicable to a wide range of developmental processes, considering there are core regulatory networks governing hematopoiesis^75^, neural development^76,77^, and other developmental processes^78^.

## Methods

We use three different models, the coarse-grained ODE model, the Boolean network model, and the SDE model, to quantify the dynamic properties of the core endogenous network. The coarse-grained ODE model quantifies the stable states, transition/hyper-transition states, and their topological connection relationships. The Boolean network model only gives the stable states information, however, the model is less dependent on the parameters. In the SDE model, the MPPs which favor the transitions can be calculated. The details of these models and analyses are described here.

### Coarse-grained ODE model

The detailed regulations of gene expression are highly complex, and the real regulatory parameters are lacked. However, to obtain the raw estimation of the essential dynamic property of the regulatory network, it is unnecessary to focus on the details of the complicated regulation. Here we use a coarse-grained model to describe the regulatory dynamics by applying the Hill-function.

The Hill-function is frequently used to model the kinetics of the enzyme-catalyzed gene transcriptions. The Hill-function is given by

1 $$H(x)=\left\{\begin{array}{c}\frac{{x}^{n}}{{K}^{n}+{x}^{n}},\,x\in activator\\ \frac{{K}^{n}}{{K}^{n}+{x}^{n}},\,x\in inhibitor\end{array}\right.,$$

where *n* is the Hill coefficient that determines the steepness of *H*, and *K* is the dissociation constant which is equal to the value of *x* at which *H* reaches its half maximum.

Generally, a gene has multiple regulators. Using the Hill-function, we approximate the expression dynamics of the target genes regulated by multiple regulators in the network by a set of ordinary differential equations (ODEs)

2 $${\dot{x}}_{i}={\eta }_{j}\cdot \frac{{\varSigma }_{u\in activators}{x}_{u}^{n}}{{K}_{i}^{n}+{\varSigma }_{u\in activators}{x}_{u}^{n}}\cdot \frac{{K}_{i}^{n}}{{K}_{i}^{n}+{\varSigma }_{r\in inhibitors}{x}_{r}^{n}}-{\tau }_{i}\cdot {x}_{i},$$

were *x*_*i*_ represents the concentration of the gene *i* in the network. *η*_*i*_ is the production rate and *τ*_*i*_ is the decay rate.

A normalization approach is used here, by which the concentrations of the TFs are scaled to [0, 1], where “1” represents the highest expression and “0” represents no expression. Here we choose *η* *τ* = 1. Besides, we assume *H*(*x*) reaches the half level of its maximum if the expression of the activator/inhibitor is at half level, which leads to *K* = 0.5. Further, to ensure the value of the Hill-function is able to vary in a wide range of values from 0 to 1, *n* should be large enough. Here, the empirical value for the network is *n* ≥ 4. With the increase of *n*, the Hill-function converges to a step function. Finally, the equations are simplified into the following form

3 $${\dot{x}}_{i}=\frac{{2}^{n}\times {\sum }_{u\in activators}{x}_{u}^{n}}{1+{2}^{n}\times {\sum }_{u\in activators}{x}_{u}^{n}}\cdot \frac{1}{1+{2}^{n}\times {\sum }_{r\in inhibitors}{x}_{r}^{n}}-{x}_{i}.$$

Denote

4 $${f}_{i}=\frac{{2}^{n}\times {\sum }_{u\in activators}{x}_{u}^{n}}{1+{2}^{n}\times {\sum }_{u\in activators}{x}_{u}^{n}}\cdot \frac{1}{1+{2}^{n}\times {\sum }_{r\in inhibitors}{x}_{r}^{n}}-{x}_{i}.$$

then the Jacobian matrix is expressed as

5 $$J(f)=\frac{\partial ({f}_{1},{f}_{2},\ldots )}{\partial ({x}_{1},{x}_{2},\ldots )}.$$

The equilibrium points are the positions at which the expressions do not change, which satisfy *f*(*x*) = 0. The equilibrium points are further classified into stable states (attractors) or transition states/hyper-transition states (saddles) by the eigenvalue characteristics of their Jacobian matrice. If the real parts of the eigenvalues of the Jacobian matrix at the equilibrium point are all negative, then the equilibrium point is identified stable state, and is denoted by S. If only one of the eigenvalues has positive real part, the equilibrium point is identified as transition state and is denoted by T. If two or more eigenvalues have positive real parts, the equilibrium point is identified as hyper-transition state, and is denoted by H. No more complicated situations, for example, all zero of the real parts, occur. The equilibrium points are obtained using the multivariate Newton method. No limit cycle is found in the ODE model using the Euler method.

### Topological connection analysis

An algorithm is designed to find the topological connections of the stable states, transition states, and hyper-transition states. Small perturbations at the transition/hyper-transition states can trigger the cell going to other states along with some specific trajectories. We generate the perturbation vectors by linear combinations of the eigenvectors of the Jacobian matrix corresponding to the eigenvalues that have positive real parts. That is, the perturbations are performed on the unstable subspace^79^ of the transition/hyper-transition states. And the amplitude of the perturbation Δ*p* is restricted by the inner product 〈Δ*p*, Δ*p*〉 < *δ*_1_, where *δ*_1_ = 0.25 × 10^−8^. Under the restricted perturbations, the trajectories were numerically calculated, and their connecting states were found. A state *x*_0_ is defined as a reachable state by a trajectory {*x*_*i*_} if there exist *x*_*i*_, satisfying 〈*x*_0_ − *x*_*i*_, *x*_0_ − *x*_*i*_〉 < *δ*_2_, where *δ*_2_ = 1 × 10^−6^.

### Boolean network model

The Boolean network model was used to obtain stable states in the core endogenous network. In the Boolean network modeling, “0/1” are two binary states representing expression “off/on”. Given the state *S*(*t*) at time *t*, the state *X*(*t* + 1) at time *t* + 1 is defined as *X*(*t* + 1) = *K*[*WX*(*t*)], where *W* is the weight matrix which represents the strengths of the activation/inhibition regulations, and *K* is the threshold that returns to “0/1” binary digits. In our analysis, the inhibition regulation is considered as dominant regulation by assigning a larger weight. Generally, the evolutionary status of the factors in the network along with time is given by

6 $${x}_{i}(t+1)=\left\{\begin{array}{c}1\,\sum _{j}{w}_{ij}{x}_{j}(t) > 0\\ 0\,\sum _{j}{w}_{ij}{x}_{j}(t)\le 0\end{array}\right.,$$

where

7 $${w}_{ij}=\left\{\begin{array}{ll}0 & {x}_{j}\notin activators/inhibitors\,of\,{x}_{i}\\ 1 & \,{x}_{j}\in activators\,of\,{x}_{i}\\ -100 & \,{x}_{j}\in inhibitors\,of\,{x}_{i}\,\end{array}\right..$$

The stable states satisfy *X*(*t*) = *X*(*t* + 1). All possible initial states, including 2^8^(256) different states, were generated during simulation.

### MPP under A-type stochastic integration

For a set of A-type stochastic equations

8 $$dx=f(x)dt+B(x)\ast dW(t),$$

where * represents A-type stochastic integration, and $B{B}^{T}=2{\epsilon }D(x)$. $\epsilon$ is the noise strength which plays the role of temperature. They can be transformed into Ito-type SDE form^80^,

9 $$\begin{array}{rcl}dx & = & -[D(x)+Q(x)]\nabla U(x)dt+B(x)\ast dW(t)\\  & = & \{-[D(x)+Q(x)]\nabla U(x)+{\epsilon }\Delta f(x)\}dt+B(x)dW(t),\end{array}$$

where *D*(*x*) and *Q*(*x*) are symmetric and anti-symmetric matrix respectively. *U(x)* is the A-type potential, $\Delta {f}_{i}(x)=\sum _{j}{\partial }_{j}[{D}_{ij}(x)+{Q}_{ij}(x)]$^80^.

The steady state distribution under A-type integration is

10 $${\rho }_{ss}(x)=exp\left[\,-\frac{U(x)}{{\epsilon }}\right].$$

Moreover, *U(x)* can be obtained by

11 $$U(x)=min\,S(x),$$

where *S* is the action functional given by^72^

12 $${S}_{{T}_{1}{T}_{2}}(x)=\frac{1}{4}{\int }_{{T}_{1}}^{{T}_{2}}\langle \dot{x}-f(x),{D}^{-1}[\dot{x}-f(x)]\rangle ds\,.$$

The most probable path is *x* which minimizes *S*(*x*).

### Computation of the MPP

The following discretization scheme is used in the computation. Given the time interval [*T*_1_, *T*_2_] of the trajectory, the time interval is divided into *N* equal subintervals to form a mesh,

13 $${T}_{1}={t}_{1} < {t}_{2} < \ldots  < {t}_{N+1}={T}_{2}\,,$$

*D* is set as identity matrix *I*. The action functional of path *x*(*t*) is approximated by the discretization,

14 $${S}_{{T}_{1}{T}_{2}}(x(t))=\frac{1}{4}\Delta t\mathop{\sum }\limits_{k=1}^{N}\mathop{\sum }\limits_{i=1}^{M}{\Vert \frac{{x}_{i}^{k+1}-{x}_{i}^{k}}{\Delta t}-\frac{{f}_{i}^{k+1}+{f}_{i}^{k}}{2}\Vert }^{2}.$$

The minima of the action functional are found using fminunc in MATLAB. The line segments connecting the initial states and terminal states are used as initial paths. *T* = 10 and *N* = 100 were used. We checked that larger *T* and *N* don’t significantly change the values with deviations smaller than 0.01, indicating the convergence of the results.

### Single-cell expression data analysis

The single-cell gene expression datasets were used to validate the model predictions. The predicted core TF expression statuses of pancreatic cell states were used as a reference to classify the cell types. The heatmaps were generated using R software. Package pheatmap and tsne were used. Exp = Count + 1 when analyzing murine pancreatic cell dataset, and Exp = TPM + 1 when analyzing the adult acinar cell dataset and the mouse islet endocrine α/β cell dataset.

## Supplementary information

Supplementary Materials.

## Data Availability

The accession number for the scRNA-seq data of murine pancreatic cells is GEO: GSE101099. The accession number for the scRNA-seq data of mouse islet endocrine α/β cells is GEO: GSE87375. The accession number for the scRNA-seq data of adult acinar cells is GEO: GSE80032. The single-cell data of the hESC model^29^ are provided by Anne Grapin-Botton.
