# Supplementary material for: Adaptive Landscape Shaped by Core Endogenous Network Coordinates Complex Early Progenitor Fate Commitments in Embryonic Pancreas
Source: Sci Rep. 2020 Jan 24;10:1112. doi: 10.1038/s41598-020-57903-0 (PMC6981170; doi:10.1038/s41598-020-57903-0)
Supplement: Supplementary file 1 — Supplementary Materials. [file 41598_2020_57903_MOESM1_ESM.pdf]

Supplementary Information

Adaptive Landscape Shaped by Core Endogenous Network Coordinates Complex Early Progenitor Fate Commitments in Embryonic Pancreas

Junqiang Wang[1], Ruoshi Yuan[2], Xiaomei Zhu[3], and Ping Ao[1,3,4,5*]

[1]Key Laboratory of Systems Biomedicine (Ministry of Education), Shanghai Center for Systems Biomedicine, Shanghai Jiao Tong University, Shanghai, China

[2]School of Biomedical Engineering, Shanghai Jiao Tong University, Shanghai, China

[3]Shanghai Center for Quantitative Life Sciences and Physics Department, Shanghai University, Shanghai, China

[4]School of Biomedical Engineering, Shanghai Jiao Tong University, Shanghai, China

[5]State Key Laboratory for Oncogenes and Related Genes, Shanghai Cancer Institute, Shanghai Jiao Tong University School of Medicine, Shanghai, China

* Correspondence to: Ping Ao, E-mail: aoping@sjtu.edu.cn

	PDX1	PTF1A	NKX6.1	SOX9	HES1	NGN3	ARX	PAX4	References
Acinar/Tip	-	+	-	-	-	-	-	-	[1,2]
Trunk	-	-	+	+	+	-	-	-	[2]
Iα	+	-	+	-	-	+	-	+	[3]
Iβ	-	-	-	-	-	+	+	-	[3]
Ductal	-	-	-	+	+	-	-	-	[2]
MP	+	+	+	+	+	-	-	-	[1]
EEP	+	-	+	+	+	+	-	-	[4,5]

Supplementary Table S1. Experimental gene expression patterns at the core network level in different pancreatic cells. Experimental observations of the core TF expression statuses in known pancreas cell types are summarized here. "+" represents expression "on", and "-" represents expression "off".

	H1	H2	H3	H4	H5	H6	H7	H8
PDX1	0.92	0.50	0.50	0.50	0.50	0.50	0.00	0.00
PTF1A	0.61	0.53	0.53	0.00	0.92	0.54	0.51	0.00
NKX6.1	0.28	0.23	0.22	0.93	0.04	0.21	0.00	0.50
SOX9	0.52	0.92	0.51	0.54	0.50	0.00	0.51	0.52
HES1	0.53	0.92	0.52	0.58	0.50	0.00	0.51	0.55
NGN3	0.21	0.07	0.19	0.26	0.05	0.21	0.16	0.23
ARX	0.03	0.00	0.02	0.06	0.00	0.03	0.01	0.04
PAX4	0.03	0.00	0.02	0.06	0.00	0.03	0.01	0.04

Supplementary Figure S1. Hyper-transition states in the core endogenous network ($n = 4$). Each column represents one state. The same states were found when using 10000, 100000, and 1000000 random initial values. “1” represents the highest expression and “0” represents no expression.

a

	T1	T2	T3	T4	T5	T6	T7	T8	T9	T10	T11	T12	T13	T14	T15	T16
S1	472	0	488	0	517	0	0	0	0	0	0	0	0	0	0	0
S2	528	487	0	518	0	0	0	0	0	0	0	0	0	0	0	0
S3	0	0	0	482	0	505	461	0	0	0	0	0	0	0	0	0
S4	0	0	0	0	0	495	0	0	0	0	0	0	0	496	507	0
S5	0	0	0	0	483	0	0	0	503	507	0	0	0	0	0	0
S6	0	0	0	0	0	0	0	0	0	0	0	513	0	0	0	0
S7	0	0	0	0	0	0	0	0	0	0	0	487	0	0	0	0
S8	0	0	0	0	0	0	539	0	0	493	508	0	0	0	0	0
S9	0	513	0	0	0	0	0	498	0	0	0	0	0	504	0	0
S10	0	0	0	0	0	0	0	0	497	0	0	0	0	0	0	496
S11	0	0	0	0	0	0	0	0	0	0	492	0	482	0	493	504

b

	H1	H2	H3	H4	H5	H6	H7	H8
S1	88	168	40	90	0	0	0	0
S2	424	360	84	0	215	0	0	0
S3	0	119	348	0	299	0	446	0
S4	0	0	50	0	199	208	67	0
S5	0	345	13	426	0	0	0	265
S6	0	0	0	0	0	0	0	0
S7	0	0	0	0	0	0	0	0
S8	0	8	11	0	0	0	58	248
S9	65	0	27	0	287	334	0	0
S10	0	0	4	82	0	0	0	253
S11	0	0	15	0	0	0	429	234

c

	T1	T2	T3	T4	T5	T6	T7	T8	T9	T10	T11	T12	T13	T14	T15	T16
T1	0	0	0	0	0	0	0	0	0	0	0	0	0	0	0	0
T2	0	0	0	0	0	0	0	0	0	0	0	0	0	0	0	0
T3	0	0	0	0	0	0	0	0	0	0	0	0	0	0	0	0
T4	0	0	0	0	0	0	0	0	0	0	0	0	0	0	0	0
T5	0	0	0	0	0	0	0	0	0	0	0	0	0	0	0	0
T6	0	0	0	0	0	0	0	0	0	0	0	0	0	0	0	0
T7	0	0	0	0	0	0	0	0	0	0	0	0	0	0	0	0
T8	0	0	0	0	0	0	0	0	0	0	0	0	0	0	0	0
T9	0	0	0	0	0	0	0	0	0	0	0	0	0	0	0	0
T10	0	0	0	0	0	0	0	0	0	0	0	0	0	0	0	0
T11	0	0	0	0	0	0	0	0	0	0	0	0	0	0	0	0
T12	0	0	512	0	0	0	0	502	0	0	0	0	518	0	0	0
T13	0	0	0	0	0	0	0	0	0	0	0	0	0	0	0	0
T14	0	0	0	0	0	0	0	0	0	0	0	0	0	0	0	0
T15	0	0	0	0	0	0	0	0	0	0	0	0	0	0	0	0
T16	0	0	0	0	0	0	0	0	0	0	0	0	0	0	0	0

d

	H1	H2	H3	H4	H5	H6	H7	H8
T1	0	0	0	0	0	0	0	0
T2	0	0	0	0	0	0	0	0
T3	0	0	0	0	0	0	0	0
T4	0	0	0	0	0	0	0	0
T5	0	0	0	0	0	0	0	0
T6	0	0	0	0	0	0	0	0
T7	0	0	0	0	0	0	0	0
T8	0	0	0	0	0	0	0	0
T9	0	0	0	0	0	0	0	0
T10	0	0	0	0	0	0	0	0
T11	0	0	0	0	0	0	0	0
T12	461	0	418	433	0	487	0	0
T13	0	0	0	0	0	0	0	0
T14	0	0	0	0	0	0	0	0
T15	0	0	0	0	0	0	0	0
T16	0	0	0	0	0	0	0	0

Supplementary Figure S2. Topological connections of stable states, transition states, and hyper-transition states ($n = 4$). (**a**) Connections between stable states and transition states. (**b**) Connections between hyper-transition states and stable states. (**c**) Connections between transition states and transition states. (**d**) Connections between hyper-transition states and transition states. 1000 perturbations were generated at each transition or hyper-transition state. The numbers indicate the counts of the trajectories reaching the states.

a

	S1	S2	S3	S4	S5	S6	S7	S8	S9	S10	S11
PDX1	0.96	0.96	0.00	0.00	0.00	0.98	0.00	0.00	0.96	0.00	0.00
PTF1A	0.00	0.96	0.96	0.96	0.00	0.00	0.00	0.00	0.96	0.00	0.00
NKX6.1	0.98	0.03	0.00	0.00	0.96	0.98	0.00	0.00	0.03	0.96	0.00
SOX9	0.96	0.96	0.96	0.00	0.96	0.00	0.00	0.96	0.00	0.00	0.00
HES1	0.96	0.96	0.96	0.00	0.96	0.00	0.00	0.96	0.00	0.00	0.00
NGN3	0.04	0.02	0.02	0.00	0.03	0.98	0.96	0.03	0.03	0.00	0.00
ARX	0.00	0.00	0.00	0.00	0.00	0.03	0.96	0.00	0.00	0.00	0.00
PAX4	0.00	0.00	0.00	0.00	0.00	0.97	0.03	0.00	0.00	0.00	0.00

b

	T1	T2	T3	T4	T5	T6	T7	T8	T9	T10	T11	T12	T13	T14	T15	T16	T17	T18
PDX1	0.96	0.96	0.96	0.50	0.50	0.00	0.00	0.96	0.00	0.00	0.00	0.25	0.27	0.50	0.00	0.00	0.27	0.03
PTF1A	0.56	0.96	0.00	0.96	0.00	0.96	0.50	0.58	0.00	0.00	0.00	0.00	0.00	0.96	0.50	0.00	0.00	0.00
NKX6.1	0.34	0.03	0.98	0.02	0.97	0.00	0.00	0.31	0.96	0.50	0.00	0.02	0.04	0.02	0.00	0.50	0.79	0.00
SOX9	0.96	0.50	0.53	0.96	0.96	0.50	0.96	0.00	0.51	0.96	0.51	0.00	0.00	0.00	0.00	0.00	0.00	0.00
HES1	0.96	0.50	0.58	0.96	0.96	0.50	0.96	0.00	0.52	0.96	0.52	0.00	0.00	0.00	0.00	0.00	0.00	0.00
NGN3	0.03	0.03	0.31	0.02	0.03	0.02	0.03	0.31	0.24	0.03	0.24	0.96	0.49	0.02	0.00	0.00	0.49	0.50
ARX	0.00	0.00	0.08	0.00	0.00	0.00	0.00	0.08	0.02	0.00	0.02	0.49	0.34	0.00	0.00	0.00	0.34	0.47
PAX4	0.00	0.00	0.08	0.00	0.00	0.00	0.00	0.08	0.02	0.00	0.02	0.49	0.42	0.00	0.00	0.00	0.42	0.29

c

	H1	H2	H3	H4	H5	H6	H7	H8	H9	H10
PDX1	0.96	0.50	0.50	0.50	0.50	0.50	0.00	0.00	0.27	0.17
PTF1A	0.57	0.51	0.51	0.00	0.96	0.52	0.50	0.00	0.00	0.00
NKX6.1	0.33	0.24	0.24	0.97	0.02	0.23	0.00	0.50	0.56	0.00
SOX9	0.51	0.96	0.50	0.52	0.50	0.00	0.50	0.51	0.00	0.00
HES1	0.52	0.96	0.51	0.54	0.50	0.00	0.50	0.52	0.00	0.00
NGN3	0.23	0.03	0.21	0.27	0.02	0.23	0.17	0.24	0.49	0.50
ARX	0.02	0.00	0.01	0.05	0.00	0.02	0.00	0.02	0.34	0.39
PAX4	0.02	0.00	0.01	0.05	0.00	0.02	0.00	0.02	0.42	0.39

Supplementary Figure S3. Stable states, transition states, and hyper-transition states in the core endogenous network ($n = 5$). (**a**) Stable states. (**b**) Transition states. (**c**) Hyper-transition states. The same states were found when using 10000, 100000, and 1000000 random initial values. "1" represents the highest expression and "0" represents no expression.

a

	S1	S2	S3	S4	S5	S6	S7	S8	S9	S10	S11
PDX1	0.98	0.98	0.00	0.00	0.00	0.99	0.00	0.00	0.98	0.00	0.00
PTF1A	0.00	0.98	0.98	0.98	0.00	0.00	0.00	0.00	0.98	0.00	0.00
NKX6.1	0.99	0.02	0.00	0.00	0.98	0.99	0.00	0.00	0.02	0.98	0.00
SOX9	0.98	0.98	0.98	0.00	0.98	0.00	0.00	0.98	0.00	0.00	0.00
HES1	0.98	0.98	0.98	0.00	0.98	0.00	0.00	0.98	0.00	0.00	0.00
NGN3	0.02	0.01	0.01	0.00	0.02	0.99	0.98	0.02	0.02	0.00	0.00
ARX	0.00	0.00	0.00	0.00	0.00	0.02	0.98	0.00	0.00	0.00	0.00
PAX4	0.00	0.00	0.00	0.00	0.00	0.98	0.02	0.00	0.00	0.00	0.00

b

	T1	T2	T3	T4	T5	T6	T7	T8	T9	T10	T11	T12	T13	T14	T15	T16	T17	T18
PDX1	0.98	0.98	0.98	0.50	0.50	0.00	0.00	0.98	0.00	0.00	0.00	0.25	0.32	0.50	0.00	0.00	0.32	0.01
PTF1A	0.55	0.98	0.00	0.00	0.98	0.98	0.50	0.56	0.00	0.00	0.00	0.00	0.00	0.98	0.50	0.00	0.00	0.00
NKX6.1	0.37	0.02	0.99	0.98	0.01	0.00	0.00	0.34	0.98	0.50	0.00	0.01	0.06	0.01	0.00	0.50	0.91	0.00
SOX9	0.98	0.50	0.52	0.98	0.98	0.50	0.98	0.00	0.50	0.98	0.50	0.00	0.00	0.00	0.00	0.00	0.00	0.00
HES1	0.98	0.50	0.56	0.98	0.98	0.50	0.98	0.00	0.51	0.98	0.51	0.00	0.00	0.00	0.00	0.00	0.00	0.00
NGN3	0.02	0.02	0.33	0.02	0.01	0.01	0.02	0.34	0.24	0.02	0.24	0.98	0.49	0.01	0.00	0.00	0.49	0.50
ARX	0.00	0.00	0.07	0.00	0.00	0.00	0.00	0.08	0.01	0.00	0.01	0.50	0.32	0.00	0.00	0.00	0.32	0.49
PAX4	0.00	0.00	0.07	0.00	0.00	0.00	0.00	0.08	0.01	0.00	0.01	0.50	0.44	0.00	0.00	0.00	0.44	0.27

c

	H1	H2	H3	H4	H5	H6	H7	H8	H9	H10	H11	H12
PDX1	0.98	0.50	0.50	0.50	0.50	0.50	0.00	0.00	0.32	0.16	0.16	0.16
PTF1A	0.55	0.50	0.51	0.00	0.98	0.51	0.50	0.00	0.00	0.00	0.00	0.00
NKX6.1	0.36	0.25	0.24	0.98	0.01	0.24	0.00	0.50	0.51	0.00	0.71	0.61
SOX9	0.50	0.98	0.50	0.51	0.50	0.00	0.50	0.50	0.00	0.00	0.00	0.00
HES1	0.51	0.98	0.50	0.53	0.50	0.00	0.50	0.51	0.00	0.00	0.00	0.00
NGN3	0.25	0.02	0.21	0.29	0.01	0.24	0.17	0.24	0.49	0.50	0.50	0.50
ARX	0.02	0.00	0.01	0.03	0.00	0.01	0.00	0.01	0.32	0.40	0.40	0.40
PAX4	0.02	0.00	0.01	0.03	0.00	0.01	0.00	0.01	0.44	0.40	0.40	0.40

Supplementary Figure S4. Stable states, transition states, and hyper-transition states in the core endogenous network (n = 6). (**a**) Stable states. (**b**) Transition states. (**c**) Hyper-transition states. The same states were found when using 10000, 100000, and 1000000 random initial values. "1" represents the highest expression and "0" represents no expression.

a

	S1	S2	S3	S4	S5	S6	S7	S8	S9	S10	S11
PDX1	0.99	0.99	0.00	0.00	0.00	1.00	0.00	0.00	0.99	0.00	0.00
PTF1A	0.00	0.99	0.99	0.99	0.00	0.00	0.00	0.00	0.99	0.00	0.00
NKX6.1	1.00	0.01	0.00	0.00	0.99	1.00	0.00	0.00	0.01	0.99	0.00
SOX9	0.99	0.99	0.99	0.00	0.99	0.00	0.00	0.99	0.00	0.00	0.00
HES1	0.99	0.99	0.99	0.00	0.99	0.00	0.00	0.99	0.00	0.00	0.00
NGN3	0.01	0.00	0.00	0.00	0.01	1.00	0.99	0.01	0.01	0.00	0.00
ARX	0.00	0.00	0.00	0.00	0.00	0.01	0.99	0.00	0.00	0.00	0.00
PAX4	0.00	0.00	0.00	0.00	0.00	0.99	0.01	0.00	0.00	0.00	0.00

b

	T1	T2	T3	T4	T5	T6	T7	T8	T9	T10	T11	T12	T13	T14	T15	T16	T17*	T18
PDX1	0.99	0.99	0.99	0.50	0.50	0.00	0.00	0.99	0.00	0.00	0.00	0.25	0.35	0.50	0.00	0.00	0.35	0.01
PTF1A	0.53	0.99	0.00	0.00	0.99	0.99	0.50	0.54	0.00	0.00	0.00	0.00	0.00	0.99	0.50	0.00	0.00	0.00
NKX6.1	0.38	0.01	1.00	0.99	0.00	0.00	0.00	0.35	0.99	0.50	0.00	0.00	0.95	0.00	0.00	0.50	0.07	0.00
SOX9	0.99	0.50	0.51	0.99	0.99	0.50	0.99	0.00	0.50	0.99	0.50	0.00	0.00	0.00	0.00	0.00	0.00	0.00
HES1	0.99	0.50	0.55	0.99	0.99	0.50	0.99	0.00	0.50	0.99	0.50	0.00	0.00	0.00	0.00	0.00	0.00	0.00
NGN3	0.01	0.01	0.34	0.01	0.00	0.00	0.01	0.35	0.24	0.01	0.24	0.99	0.49	0.00	0.00	0.00	0.49	0.50
ARX	0.00	0.00	0.06	0.00	0.00	0.00	0.00	0.08	0.01	0.00	0.01	0.50	0.32	0.00	0.00	0.00	0.32	0.50
PAX4	0.00	0.00	0.06	0.00	0.00	0.00	0.00	0.08	0.01	0.00	0.01	0.50	0.45	0.00	0.00	0.00	0.45	0.26

c

	H1	H2	H3	H4	H5	H6	H7	H8	H9	H10	H11	H12
PDX1	0.99	0.50	0.50	0.50	0.50	0.50	0.00	0.00	0.15	0.15	0.15	0.35
PTF1A	0.54	0.50	0.50	0.00	0.99	0.50	0.50	0.00	0.00	0.00	0.00	0.00
NKX6.1	0.38	0.25	0.25	0.99	0.00	0.25	0.00	0.50	0.56	0.00	0.78	0.50
SOX9	0.50	0.99	0.50	0.51	0.50	0.00	0.50	0.50	0.00	0.00	0.00	0.00
HES1	0.51	0.99	0.50	0.52	0.50	0.00	0.50	0.50	0.00	0.00	0.00	0.00
NGN3	0.27	0.01	0.22	0.30	0.01	0.25	0.17	0.24	0.50	0.50	0.50	0.49
ARX	0.01	0.00	0.00	0.03	0.00	0.01	0.00	0.01	0.41	0.41	0.41	0.32
PAX4	0.01	0.00	0.00	0.03	0.00	0.01	0.00	0.01	0.41	0.41	0.41	0.45

Supplementary Figure S5. Stable states, transition states, and hyper-transition states in the core endogenous network ($n = 7$). (**a**) Stable states. (**b**) Transition states. (**c**) Hyper-transition states. The same stable states were found when using 10000, 100000, and 1000000 random initial values. "1" represents the highest expression and "0" represents no expression. T17* differs from T17 by having a significant low expression level of NKX6.1.

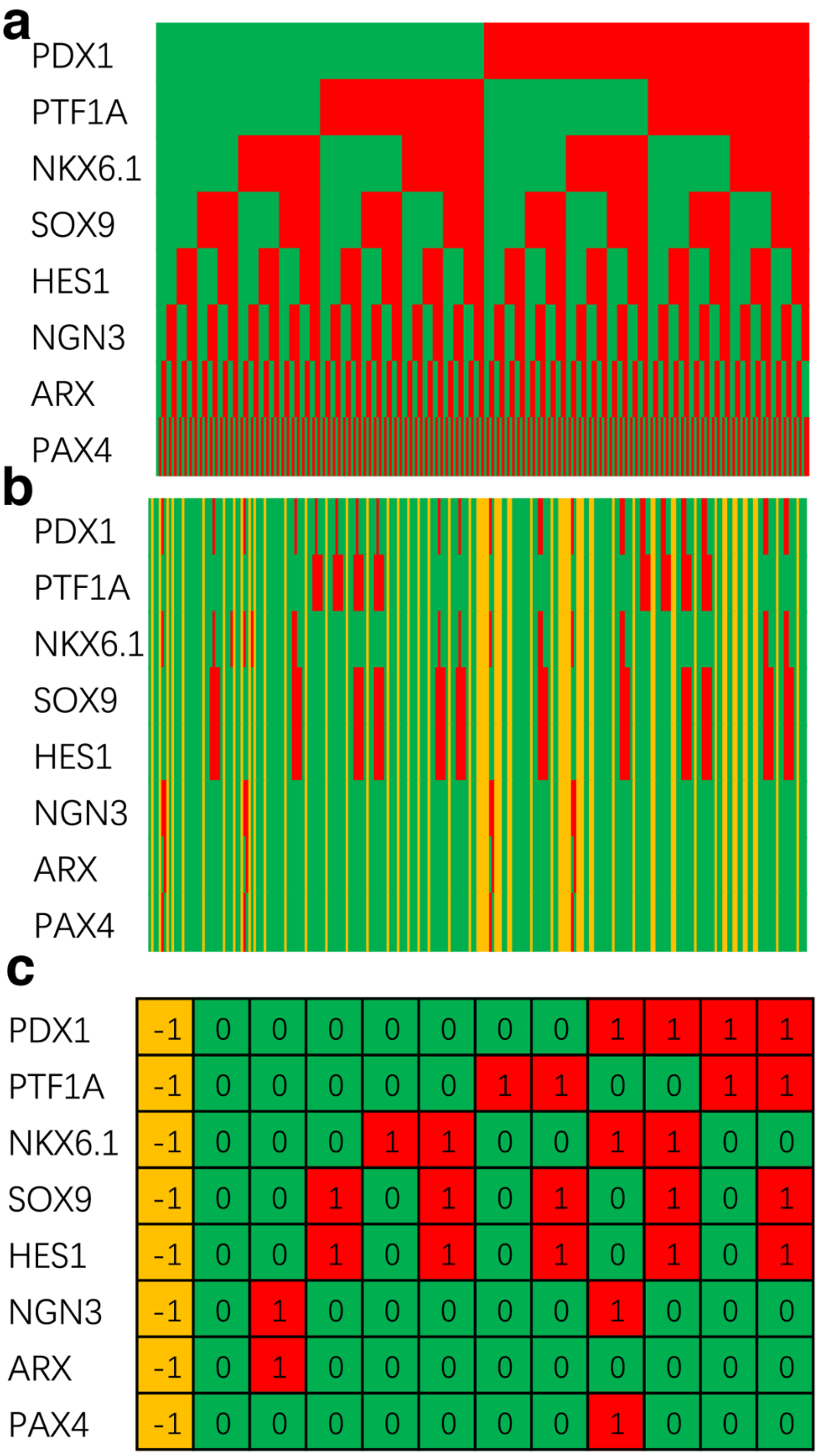

Supplementary Figure S6. Boolean network modeling results of the core endogenous network. (**a**) All of the possible initial states ($2^8 = 256$) in the Boolean network. Each column represents one initial state. (**b**) The end states corresponding to the initial states. (**c**) All of the non-redundant end states in (**b**). “1” represents expression “on”, and is colored by red. “0” represents expression “off”, and is colored by green. While “-1” represents the non-convergent point statuses and is colored by orange in (**b**) and (**c**).

a

	S1	S2	S5	S6	S7	S8	S9	S10	S11
PDX1	0.92	0.96	0.00	0.96	0.00	0.00	0.96	0.00	0.00
PTF1A	0.00	0.92	0.00	0.00	0.00	0.00	0.92	0.00	0.00
NKX6.1	0.96	0.08	0.92	0.96	0.00	0.00	0.08	0.92	0.00
SOX9	0.92	0.92	0.92	0.00	0.00	0.92	0.00	0.00	0.00
HES1	0.92	0.92	0.92	0.00	0.00	0.92	0.00	0.00	0.00
NGN3	0.08	0.04	0.07	0.97	0.92	0.07	0.08	0.00	0.00
ARX	0.00	0.00	0.00	0.07	0.92	0.00	0.00	0.00	0.00
PAX4	0.00	0.00	0.00	0.93	0.07	0.00	0.00	0.00	0.00

b

	T1	T2	T3	T5	T8	T9	T10	T11	T12	T13	T16
PDX1	0.93	0.96	0.92	0.50	0.94	0.00	0.00	0.00	0.26	0.19	0.00
PTF1A	0.60	0.92	0.00	0.00	0.64	0.00	0.00	0.00	0.00	0.00	0.00
NKX6.1	0.30	0.08	0.96	0.93	0.26	0.92	0.50	0.00	0.04	0.02	0.50
SOX9	0.92	0.50	0.56	0.92	0.00	0.52	0.92	0.52	0.00	0.00	0.00
HES1	0.92	0.50	0.62	0.92	0.00	0.55	0.92	0.55	0.00	0.00	0.00
NGN3	0.07	0.07	0.28	0.07	0.26	0.23	0.07	0.23	0.92	0.49	0.00
ARX	0.00	0.00	0.09	0.00	0.06	0.04	0.00	0.04	0.49	0.37	0.00
PAX4	0.00	0.00	0.09	0.00	0.06	0.04	0.00	0.04	0.49	0.37	0.00

c

	H1	H4	H8
PDX1	0.94	0.50	0.00
PTF1A	0.61	0.00	0.00
NKX6.1	0.28	0.93	0.50
SOX9	0.52	0.54	0.52
HES1	0.53	0.58	0.55
NGN3	0.21	0.26	0.23
ARX	0.03	0.06	0.04
PAX4	0.03	0.06	0.04

Supplementary Figure S7. Stable states, transition states, and hyper-transition states in the core endogenous network including the activation of PDX1 by PTF1A ($n = 4$). (**a**) Stable states. (**b**) Transition states. (**c**) Hyper-transition states. The same states were found when using 10000, 100000, and 1000000 random initial values. "1" represents the highest expression and "0" represents no expression.

a

	S1	S2	S3	S4	S5	S6	S7	S8	S9	S10	S11
PDX1	0.92	0.92	0.00	0.00	0.00	0.96	0.00	0.00	0.92	0.00	0.00
PTF1A	0.00	0.92	0.92	0.92	0.00	0.00	0.00	0.00	0.92	0.00	0.00
NKX6.1	0.96	0.07	0.00	0.00	0.92	0.97	0.00	0.00	0.07	0.92	0.00
SOX9	0.92	0.92	0.92	0.00	0.92	0.00	0.00	0.92	0.00	0.00	0.00
HES1	0.92	0.92	0.92	0.00	0.92	0.00	0.00	0.92	0.00	0.00	0.00
NGN3	0.08	0.04	0.04	0.00	0.07	0.97	0.92	0.07	0.07	0.00	0.00
ARX	0.00	0.00	0.00	0.00	0.00	0.03	0.92	0.00	0.00	0.00	0.00
PAX4	0.00	0.00	0.00	0.00	0.00	0.93	0.07	0.00	0.00	0.00	0.00

b

	T1	T2	T3	T4	T5	T6	T7	T8	T9	T10	T11	T12	T13	T14	T15	T16	T01
PDX1	0.92	0.92	0.92	0.50	0.50	0.00	0.00	0.92	0.00	0.00	0.00	0.26	0.19	0.50	0.00	0.00	0.33
PTF1A	0.60	0.92	0.00	0.00	0.92	0.92	0.50	0.63	0.00	0.00	0.00	0.00	0.00	0.92	0.50	0.00	0.00
NKX6.1	0.30	0.07	0.96	0.93	0.04	0.00	0.00	0.26	0.92	0.50	0.00	0.04	0.02	0.04	0.00	0.50	0.92
SOX9	0.92	0.50	0.56	0.92	0.92	0.50	0.92	0.00	0.52	0.92	0.52	0.00	0.00	0.00	0.00	0.00	0.00
HES1	0.92	0.50	0.62	0.92	0.92	0.50	0.92	0.00	0.55	0.92	0.55	0.00	0.00	0.00	0.00	0.00	0.00
NGN3	0.07	0.07	0.28	0.07	0.04	0.04	0.07	0.26	0.23	0.07	0.23	0.92	0.49	0.04	0.00	0.00	0.44
ARX	0.00	0.00	0.01	0.00	0.00	0.00	0.00	0.06	0.00	0.00	0.04	0.49	0.37	0.00	0.00	0.00	0.03
PAX4	0.00	0.00	0.09	0.00	0.00	0.00	0.00	0.06	0.04	0.00	0.04	0.49	0.37	0.00	0.00	0.00	0.37

c

	H1	H2	H3	H4	H5	H6	H7	H8	H9
PDX1	0.92	0.50	0.50	0.50	0.50	0.50	0.00	0.00	0.32
PTF1A	0.61	0.53	0.53	0.00	0.92	0.54	0.51	0.00	0.00
NKX6.1	0.28	0.23	0.22	0.93	0.04	0.21	0.00	0.50	0.46
SOX9	0.52	0.92	0.51	0.54	0.50	0.00	0.51	0.52	0.00
HES1	0.53	0.92	0.52	0.58	0.50	0.00	0.51	0.55	0.00
NGN3	0.21	0.07	0.19	0.26	0.05	0.21	0.16	0.23	0.44
ARX	0.03	0.00	0.02	0.00	0.00	0.03	0.01	0.02	0.19
PAX4	0.03	0.00	0.02	0.06	0.00	0.03	0.01	0.04	0.38

Supplementary Figure S8. Stable states, transition states and hyper-transition states in the core endogenous network including the inhibition of ARX by NKX6.1 ($n = 4$). (**a**) Stable states. (**b**) Transition states. T01 is a novel transition state (**c**) Hyper-transition states. The same stable states were found when using 10000, 100000, and 1000000 random initial values. "1" represents the highest expression and "0" represents no expression.

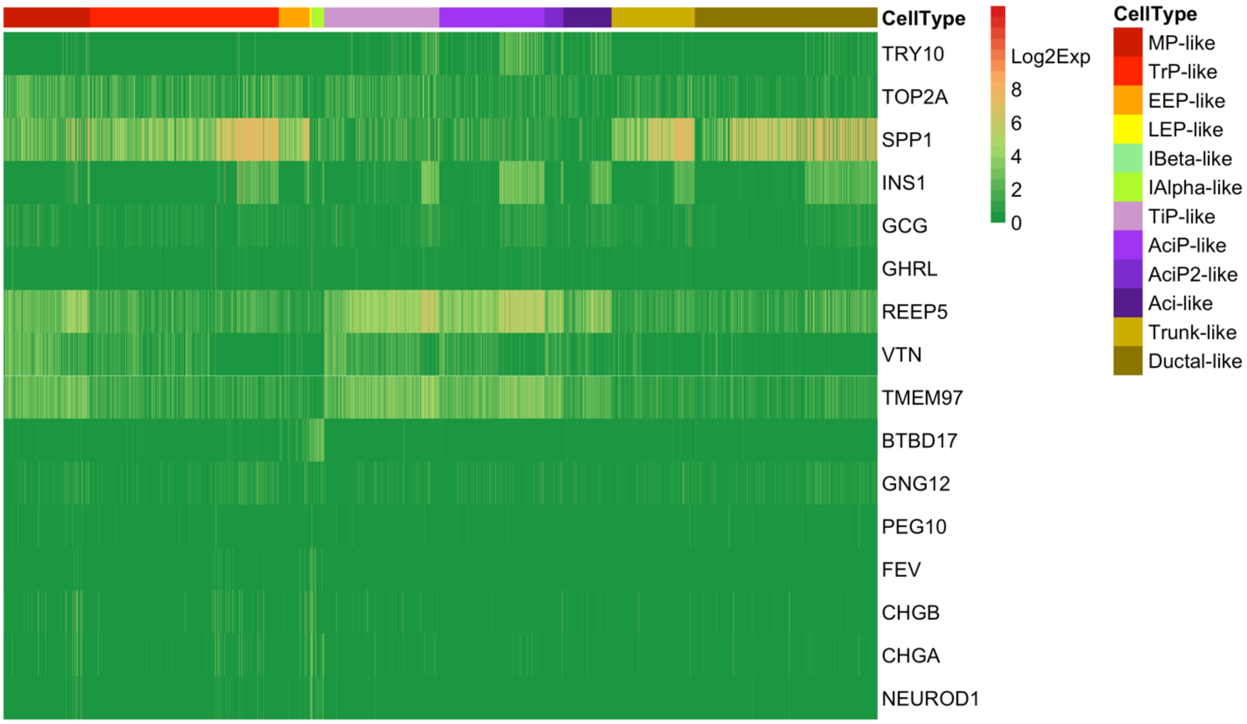

Supplementary Figure S9. Expression statuses of other reported epithelial markers in the cell types we predicted. The expressions of the reported pancreatic epithelial cell markers in the murine embryonic pancreas[6] are shown.

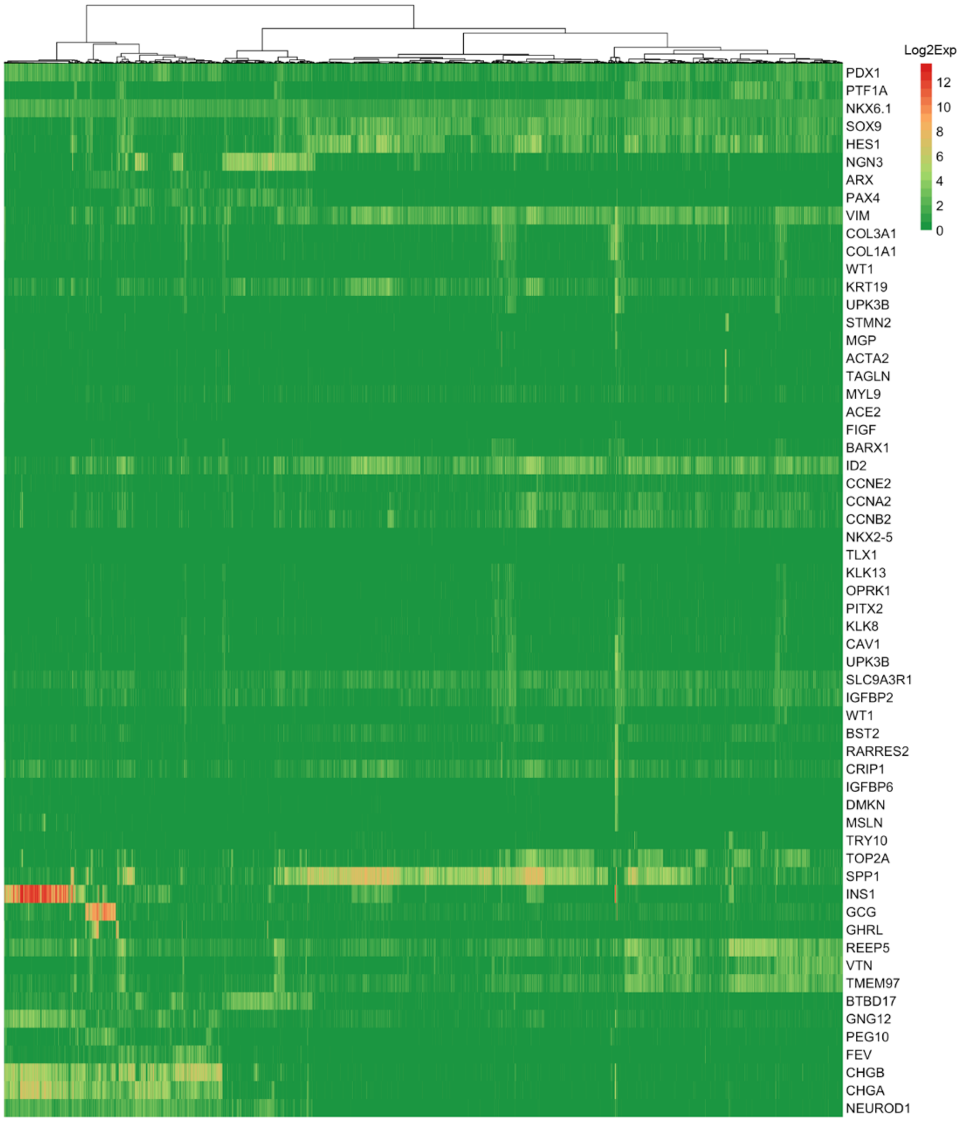

Supplementary Figure S10. Expression patterns of NKX6.1^{+} cells. The expressions of the reported pancreatic cell markers in the murine embryonic pancreas[6] are also shown. The mature β cells only express PTF1A and NKX6.1 at the core network level.

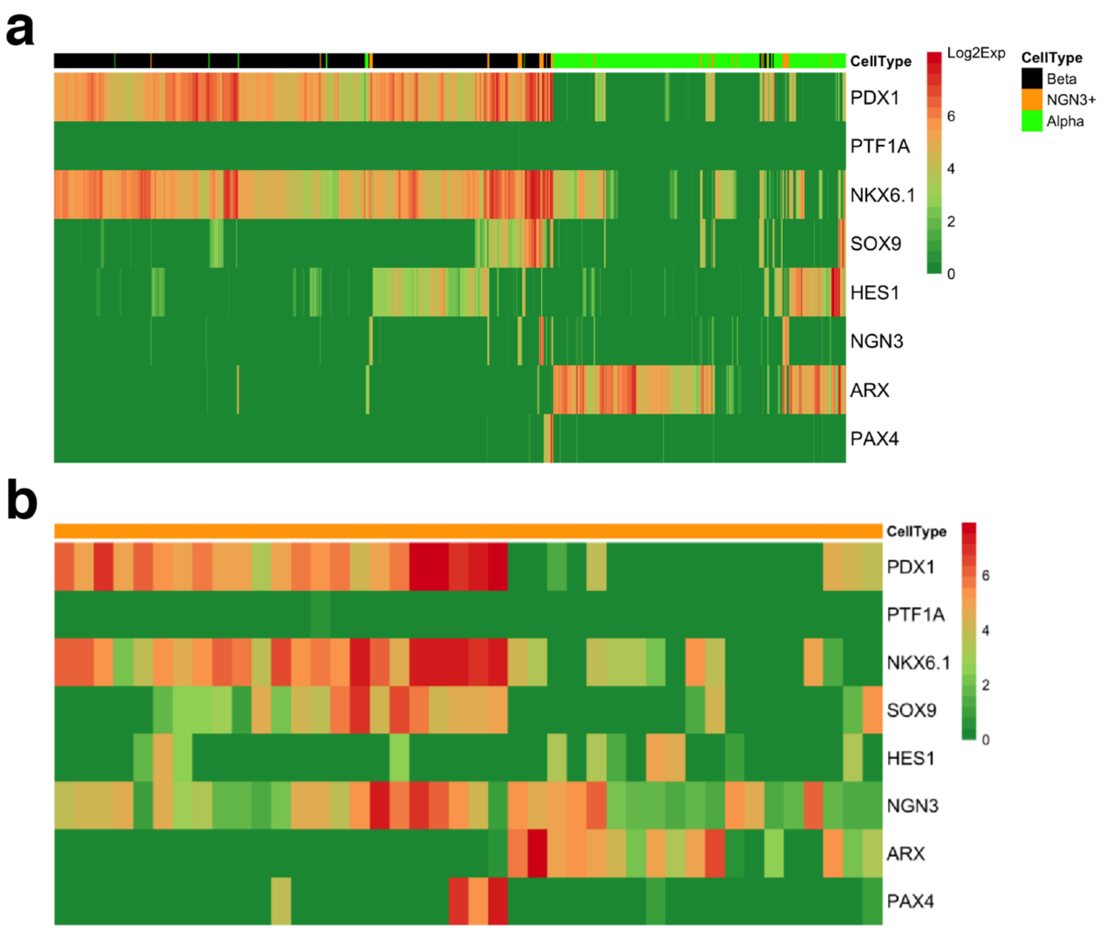

Supplementary Figure S11. Expression patterns of the mouse islet α/β cells at the core network level. (**a**) The core TF expression patterns of all mouse islet α/β cells in the dataset. Only very small proportional cells express NGN3 (log2Exp>1). (**b**) The core TF expression patterns of $NGN3^+$ cell types. Several cells show Iα-like, Iβ-like, or LEP-like expression patterns.

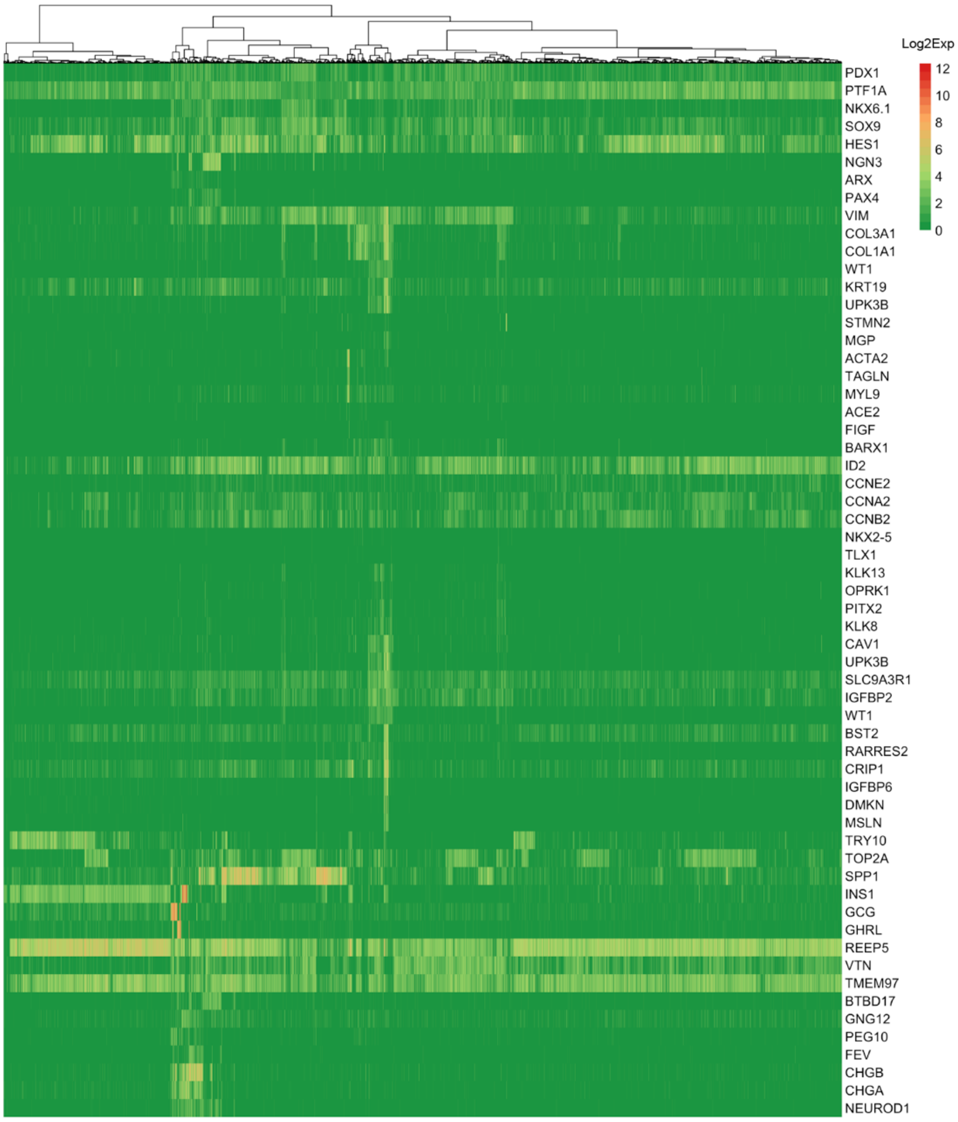

Supplementary Figure S12. Expression patterns of PTF1A$^+$ cells. The expression patterns of the reported cell markers in the murine embryonic pancreas[6] are also shown.

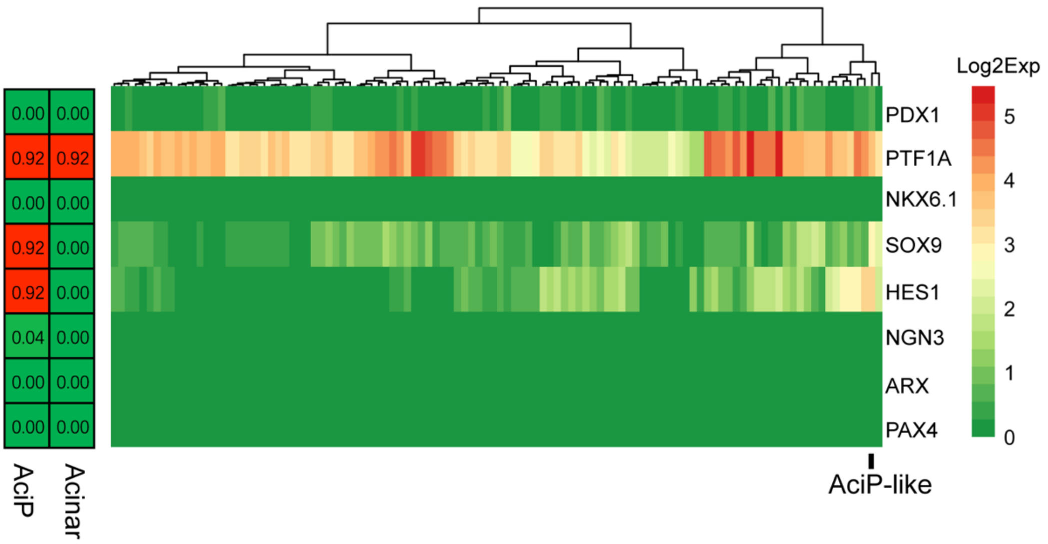

Supplementary Figure S13. Validation of predicted AciP state by analyzing the acinar scRNA-seq data. The expression patterns of acinar cells at the core network level. The SOX9$^+$STMN1$^+$ acinar progenitor-like (AciP-like) cell and the AciP state have the same expression pattern. Both of them express PTF1A, SOX9, and HES1. The model predicted states are shown on the left.

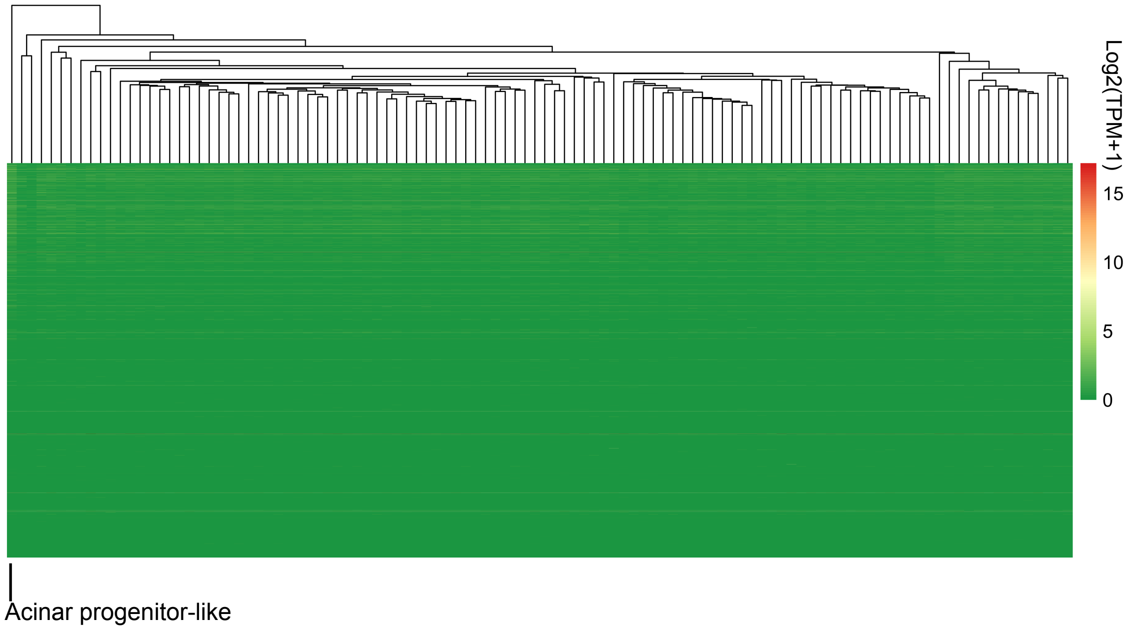

Supplementary Figure S14. Heatmap of acinar scRNA-seq data indicating the genomic expression difference. The SOX9^{+} HES1^{+} acinar progenitor-like cell differs from the acinar cells by having a long distance to the acinar cell group. The "Euclidean" clustering distance and the "complete" clustering method were used. We find that the SOX9^{+}HES1^{+} acinar progenitor-like cell has the same expression pattern as the SOX9^{+}STMN1^{+} acinar progenitor cell at the core network level. The heatmap was reproduced from the previous publication[7].

References

1 Larsen, H. L. & Grapin-Botton, A. The molecular and morphogenetic basis of pancreas organogenesis. *Semin Cell Dev Biol* **66**, 51-68, doi:10.1016/j.semcdb.2017.01.005 (2017).

2 Shih, H. P., Wang, A. & Sander, M. Pancreas organogenesis: from lineage determination to morphogenesis. *Annu Rev Cell Dev Biol* **29**, 81-105, doi:10.1146/annurev-cellbio-101512-122405 (2013).

3 Collombat, P. *et al.* Opposing actions of Arx and Pax4 in endocrine pancreas development. *Genes Dev* **17**, 2591-2603, doi:10.1101/gad.269003 (2003).

4 Jensen, J. *et al.* Independent development of pancreatic alpha- and beta-cells from neurogenin3-expressing precursors: a role for the notch pathway in repression of premature differentiation. *Diabetes* **49**, 163-176 (2000).

5 Petersen, M. B. K. *et al.* Single-Cell Gene Expression Analysis of a Human ESC Model of Pancreatic Endocrine Development Reveals Different Paths to beta-Cell Differentiation. *Stem Cell Reports* **9**, 1246-1261, doi:10.1016/j.stemcr.2017.08.009 (2017).

6 Byrnes, L. E. *et al.* Lineage dynamics of murine pancreatic development at single-cell resolution. *Nat Commun* **9**, 3922, doi:10.1038/s41467-018-06176-3 (2018).

7 Wollny, D. *et al.* Single-Cell Analysis Uncovers Clonal Acinar Cell Heterogeneity in the Adult Pancreas. *Dev Cell* **39**, 289-301, doi:10.1016/j.devcel.2016.10.002 (2016).
